# DynaMETE: a hybrid MaxEnt‐plus‐mechanism theory of dynamic macroecology

**DOI:** 10.1111/ele.13714

**Published:** 2021-03-06

**Authors:** John Harte, Kaito Umemura, Micah Brush

**Affiliations:** ^1^ The Energy and Resources Group University of California Berkeley CA 94720 USA; ^2^ The Rocky Mountain Biological Laboratory Gothic CO 81224 USA; ^3^ The Santa Fe Institute Santa Fe NM 87501 USA; ^4^ The Energy and Resources Group University of California Berkeley CA 94720 USA; ^5^ Department of Physics University of California Berkeley CA 94720 USA

**Keywords:** Abundance distribution, disturbance, DynaMETE, dynamics, macroecology, maximum entropy, mechanism, metabolic rate distribution, METE

## Abstract

The Maximum Entropy Theory of Ecology (METE) predicts the shapes of macroecological metrics in relatively static ecosystems, across spatial scales, taxonomic categories and habitats, using constraints imposed by static state variables. In disturbed ecosystems, however, with time‐varying state variables, its predictions often fail. We extend macroecological theory from static to dynamic by combining the MaxEnt inference procedure with explicit mechanisms governing disturbance. In the static limit, the resulting theory, DynaMETE, reduces to METE but also predicts a new scaling relationship among static state variables. Under disturbances, expressed as shifts in demographic, ontogenic growth or migration rates, DynaMETE predicts the time trajectories of the state variables as well as the time‐varying shapes of macroecological metrics such as the species abundance distribution and the distribution of metabolic rates over individuals. An iterative procedure for solving the dynamic theory is presented. Characteristic signatures of the deviation from static predictions of macroecological patterns are shown to result from different kinds of disturbance. By combining MaxEnt inference with explicit dynamical mechanisms of disturbance, DynaMETE is a candidate theory of macroecology for ecosystems responding to anthropogenic or natural disturbances.

## Graphical abstract

The architecture of DynaMETE, a hybrid MaxEnt‐plus‐mechanism theory of macroecology. The time evolution of Macroecological Metrics in ecosystems disturbed by a hypothesised Mechanism is derived iteratively using the MaxEnt inference procedure (step E in the figure).

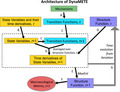

## INTRODUCTION

Ecology seeks insight into the shape and origin of patterns in the abundance, energetics and spatial distributions of taxa, across spatial scales and within different habitats. Macroecology, the study of such patterns, builds capacity for estimating species diversity from sparse data, predicting extinction rates under habitat loss and deciphering the processes governing ecosystem structure and function. (Brown 1995, Rosenzweig 1995, Gaston & Blackburn 2000, Kitzes & Shirley 2016).

Although the study of dynamic ecosystems is a rising area in ecology (Hill & Hamer^1998^; Dornelas 2010; Turner 2010; Newman 2019), macroecological theory has largely focused on patterns in quasi‐steady‐state ecosystems, ignoring trending patterns in systems undergoing rapid succession, diversification or collapse (Fisher *et al*. 2010). Empirical evidence, however, is accumulating that macroecological patterns differ between dynamic and static ecosystems (e.g. Kempton & Taylor 1974; Carey *et al*. 2006; Harte 2011; Supp *et al*. 2012; Harte & Newman 2014; Rominger *et al*. 2015; Newman *et al*. 2020). Here, we formulate and initially explore a theory, DynaMETE, to predict macroecological patterns in dynamic systems.

Our starting point is a static theory based on the maximum entropy (MaxEnt) framework (Harte 2011; Harte & Newman 2014). MaxEnt selects the flattest, and therefore least informative, probability distributions compatible with constraints imposed by prior knowledge. Bias, in the form of assumptions about the distribution that are not compelled by prior knowledge, is thereby eliminated (Jaynes 1957, 1982). The maximum entropy form of a probability distribution, *p*(*n*), is obtained by maximising its Shannon information entropy (Shannon 1948), $‐\sum_{n}p\left(n\right)\log\left(p\left(n\right)\right),$ under imposed constraints.

The MaxEnt inference procedure has been applied in many fields, including image reconstruction in medicine and forensics (Frieden, 1972; Skilling, 1984; Gull & Newton, 1986; Roussev, 2010), neural net firing patterns (Meshulam, 2017), protein folding (Steinbach et. al, 2002; Mora *et al,* 2010) and reconstruction of incomplete input–output data and other applications in economics (Golan *et al*. 1996; Golan 2018).

The MaxEnt Theory of Ecology (METE) assumes prior knowledge in the form of static state variables describing a taxonomic group of interest (e.g. plants or arthropods) in a prescribed location. In the original version of the theory, there are four state variables: area, *A*, of the ecosystem, total number of species, *S*, within the taxonomic group in that ecosystem, summed number of individuals, *N*, in those species and summed metabolic rate, *E*, of those individuals. From the constraints imposed by ratios of the state variables, METE predicts the forms of many metrics of macroecology with no adjustable parameters. Other taxonomic categories, such as genera or families, can be substituted for species.

The predicted macroecological patterns include a log‐series species abundance distribution (SAD) (Harte *et al*. 2008; Harte & Kitzes, 2014; White *et al*. 2012; but see Ulrich *et al*. 2010), the species–area relationship (SAR) (Harte *et al*. 2009), the metabolic rate distribution over individuals (MRDI) (Harte *et al*. 2008, 2017; Xiao *et al*. 2015) and a relationship between the average metabolic rate of the individuals in a species and the abundance of that species (Harte *et al*. 2008). An extension of the original theory predicts the distribution of species over broader taxonomic categories and the dependence of the abundance–metabolism relationship on the structure of the taxonomic tree (Harte *et al*. 2015).

METE’s predictions generally fail in ecosystems undergoing relatively rapid change. In particular, when state variables are changing as a consequence of succession or anthropogenic disturbance, the values of the state variables at any moment in time no longer accurately predict the shapes of the macroecological metrics at that same moment in time.

Examples of altered macroecological patterns in disturbed ecosystems abound. Moth censuses at Rothamsted reveal a log‐series SAD (as METE predicts) at less disturbed locations and a lognormal SAD, with fewer rare species, at more disturbed locations (Kempton & Taylor 1974). Supp *et al*. (2012) report that when the state variables (species richness and total abundance) are experimentally altered in small‐mammal communities, the functional form of the SAD is altered. Kunin *et al*. (2018) show that in the highly fragmented and manipulated UK, METE under‐predicts plant species richness derived from upscaling data from small plots. Franzman et al. (2021) show that in an alpine plant community, both the SAR and the SAD increasingly deviate over time from METE predictions during several years of drought stress.

In systems recovering from disturbance, macroecological patterns also change. Carey *et al*. (2006) show that the shape of the SAR in recovering subalpine vegetation plots in the aftermath of both an eruption at Mount St. Helens and a hillslope‐erosion event at Gothic CO deviated systematically from that observed in nearby undisturbed comparison plots. In the aftermath of a recent fire, Newman *et al*. (2020) observe the failure of METE for a plant community in a fire‐adapted Bishop Pine forest site in coastal California. There, the SAR in a successional post‐fire ecosystem deviates markedly from the METE prediction, in contrast to a control site that has not burned in many decades. On much longer time scales, such shifts are also occurring; for example, at younger sites in the Hawaiian Islands where arthropod diversification is occurring more rapidly, the SAD and the MRDI show deviations from static theory predictions, in contrast to older sites on the islands (Rominger *et al*. 2015).

Across the Smithsonian forest plots, systematic deviations from MaxEnt predictions appear prominently at the Barro Colorado Island site in Panama. Here, the state variables, *S* and *N*, have declined over the past 30 years, speculatively a consequence of a combination of local disturbance and the formation of Gatun Lake resulting in semi‐isolation of the created island from its metacommunity (E. Leigh, pers. comm.). The shape of the SAD at BCI is currently intermediate between a METE‐predicted log‐series and a lognormal, with proportionally more intermediate‐abundance species than observed at Smithsonian forest plots that are less disturbed, such as Cocoli and Bukit Timah, which show closer agreement with the log‐series SAD predicted by METE (Harte 2011).

The pattern of deviation of macroecological metrics from METE predictions differs across disturbed ecosystems. Whereas in some disturbance sites, the SAD trends towards a lognormal distribution (Rothamsted moths, BCI trees), in others the trend is towards a geometric distribution with a truncated tail at small *n* (alpine plant community). In some disturbance sites, the SAR deviates from the METE prediction towards a power‐law (post‐burn Bishop pine forest), while in others it deviates further from power‐law behavior (alpine plant community).

This variety of responses of macroecological patterns to disturbance challenges us to advance theory that predicts the connections. We will see that DynaMETE predicts departures of macroecological distributions from steady state that depend on the specific type of disturbance. It also predicts future trajectories of state variables describing disturbed ecosystems.

In METE, the state variables are assumed to vary so slowly that their instantaneous values suffice to derive macroecological distributions. Plausibly, in a dynamic ecosystem with rapidly changing state variables, static METE might be inadequate. Analogously in thermodynamics, where the macroscopic state variables are pressure, volume and temperature, the Boltzmann distribution of molecular kinetic energies can be derived using MaxEnt (Jaynes 1957, 1982). In an out‐of‐steady‐state ‘disturbed’ gas, such as one with inhomogeneously changing temperature, the instantaneous averaged values of pressure, volume and temperature no longer determine the instantaneous molecular energy distribution.

In DynaMETE, to the list of instantaneous constraints imposed by the values of the state variables, *S*, *N* and *E*, we add the additional constraints imposed by their first time derivatives. We then combine the MaxEnt procedure for determining least‐biased probability distributions with explicit mechanisms that drive the system from steady state. Because the dynamics depends on the time‐dependent state variables, we propose an iterative procedure for updating both constraints and macroecological distributions.

In Methods, we review METE and present the theoretical framework for DynaMETE, including how explicit mechanisms are incorporated and upscaled from individuals to the community level, and an iteration procedure for deriving predictions. In Results, we first examine predicted scaling relationships among the state variables in the static limit of DynaMETE. Then, we examine dynamic predictions of the theory near steady state. Coupled time‐differential equations for the state variables are derived and solved to reveal predicted trajectories of these variables under various perturbations. We also show predicted deviations of abundance and metabolic rate distributions in a first‐order iteration of the full theory under a variety of perturbations. In the Discussion, we summarise DynaMETE’s predictions and suggest future directions. Symbols are defined in a Glossary (Table 1).

**Table 1:** Glossary of symbols

Macrolevel state variables	
*S*	Total # species in community
*N*	Total # individuals in community
*E*	Total metabolic rate of community
*B*	Total biomass of community
*P*	Total productivity of community
*S*_m_	Total # species in metacommunity
Microlevel‐independent variables	
*n*	Abundance of a randomly selected species
ε	Metabolic rate of a randomly selected individual
*m*	Mass of an individual
Probability distributions	
*R*(*n*,ε)	Ecological structure function
ϕ(n)	Distribution of abundances over species
ψ(ε)	Distribution of metabolic rates over individuals
Lagrange multipliers and functions thereof	
$\lambda_{i}$	Index *i* runs from 1 to 5
β	$\lambda_{1}+\;\lambda_{2}$
$\beta_{m}$	β for the metacommunity
*Z*	Normalisation constant for *R*
γ(ε)	$\lambda_{1}+\;\lambda_{2}\varepsilon$
Transition rate functions	
*f*	Governs the time rate of change of abundance
*g, h*	Governs the time rate of change of metabolic rates of individuals, species
*q*	Governs the time rate of change of species richness
Transition rate parameters	
*b*_0_	A birth rate constant
*d*_0_	A death rate constant
*m*_0_	Immigration rate constant
*w*_0_, w_1_	Ontogenic growth rate constants
*w*_10_	*w*_1_/ln^2/3^(1/β)
*E*_C_	Metabolic carrying capacity of ecosystem
μ	ln(1/$\beta_{m})/S_{m}$
$\sigma_{1},\;\sigma_{2}$ speciation rate constants	
*K*	speciation saturation constant
Mathematical quantities	
γ	Euler’s constant (~0.577)
$\delta_{n,1}$	Kronecker delta function (=1 if *n* = 1; = 0 otherwise)

## METHODS

### Review of METE

The core of METE is a time‐independent ‘structure function’, *R*(*n*, ε|*S*, *N*, *E*). *R* is a joint conditional distribution over abundance, *n*, of a species, and metabolic rate, ε of an individual; *R*·dε is the probability that a species picked at random from the species pool has abundance *n*, and an individual picked at random from the species with abundance *n* has a metabolic rate in the interval (ε, ε + dε).

We use a discrete notation, with summation, not integral, signs for all variables *n*,ε, *S*, *N*, *E*, and adopt the units convention that the smallest value for the metabolic rate is = ε1, the metabolic rate of the smallest observed organism in the community (e.g. a tree with 10 mm dbh if that is the smallest tree censused). The hard (*sensu* Haegeman & Loreau 2008) constraints on the static structure function are:4

(1) $$\frac{N}{S}\;=\;\sum_{n,\varepsilon }nR\left(n,\varepsilon |S,N,E\right)$$

and

(2) $$\frac{E}{S}=\;\sum_{n,\varepsilon }n\varepsilon R\left(n,\varepsilon |S,N,E\right).$$

The MaxEnt solution (Harte *et al*. 2008) for *R*(*n*, ε|*S*,*N*,*E*) subject to these constraints is:

(3) $$R\left(n,\varepsilon |S,N,E\right)=\frac{e^{‐\lambda_{1}n}e^{{‐\lambda }_{2}n\varepsilon }}{Z}$$

where *Z* ^‐1^ is a normalisation constant (see below). The λ’s are Lagrange multipliers that are determined from the values of *S*, *N*, *E*. The quantity β = λ_1_ + λ_2_ is determined from

(4) $$\frac{S}{N}\sum_{n=1}^{N}e^{‐\beta n}=\sum_{n=1}^{N}\frac{e^{‐\beta n}}{n}.$$

The Lagrange multiplier, $\lambda_{2},$ is given by.

(5) $$\lambda_{2}=\frac{S}{E‐N}.$$

Generally, the state variables obey the inequalities S << N<< *E*, in which case β≪1 and the solution to eqn 4 is, to a good approximation,

(6) $$\frac{S}{N}\approx \beta \ln\left(\frac{1}{1‐e^{‐\beta }}\right)\approx \beta \ln\left(1/\beta \right)$$

Moreover, in that approximation,

(7) $$Z\approx \frac{\ln(1/\beta )}{\lambda_{2}},$$

In all that follows, we assume the validity of these approximations, and use =, not ≈, signs in the equations.

We note for later comparison with DynaMETE that the species abundance distribution (SAD), $\phi \left(n\right),$ obtained by summing *R* over metabolic rate, ε, is the log‐series distribution:

(8) $$\phi \left(n\right)=\frac{e^{‐\beta n}}{n∙\ln(1/\beta )}$$

and the metabolic rate distribution over individuals (the MRDI), obtained by summing *nR*S/*N* over abundance, *n*, is:

(9) $$\Psi \left(\varepsilon \right)=\frac{\beta \lambda_{2}e^{‐\gamma (\varepsilon )}}{{\left(1‐e^{‐\gamma (\varepsilon )}\right)}^{2}}$$

where

(10) $$\gamma \left(\varepsilon \right)=\;\lambda_{1}+\lambda_{2}\varepsilon$$

METE also contains a spatially‐explicit component but to focus on the essential concepts underlying DynaMETE, we ignore the spatial dimension here.

For further description of MaxEnt and METE, and software for deriving predictions, see Harte (2011); Brummer and Newman (2019); Kitzes and Wilber (2016); Rominger & Merow (2016).

### DynaMETE

DynaMETE is a hybrid theory based upon both the logic of the MaxEnt procedure and explicit mechanistic assumptions about the drivers of change. The structure function, a dynamic generalisation of eqn 3, again plays a central role linking the microlevel, described by the independent variables *n* and ε, and the macrolevel, described by state variables; from it the dynamic macroecological metrics, such as a time‐dependent SAD, can be derived. The mechanistic parent of DynaMETE is a set of transition functions that describe demographic, ontogenic growth, migration, extinction and speciation rates. They derive from analyses of individuals and populations and thus depend upon *n* and ε.

**Figure 1:**
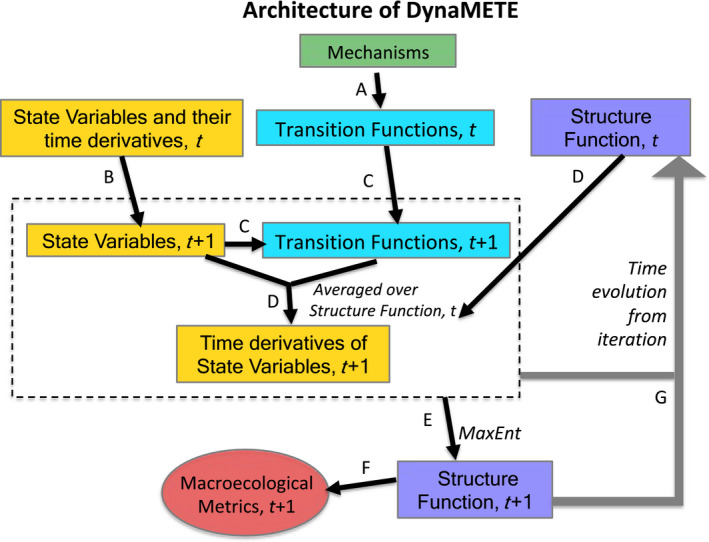
The architecture of DynaMETE. (a) Selected mechanisms are incorporated into transition functions. (b) The time derivatives of state variables update the state variables. (c) The transition functions, which depend upon the state variables are updated. (d) Updated state variables and transition functions are averaged over the prior (time *t*) structure function to update the time derivatives of the state variables. (e) Under updated constraints and transition functions (dashed box), MaxEnt updates the structure function. (f) The macroecological metrics are updated from the updated structure function. (g) Steps B–F are iterated.

To upscale from the time derivatives of the microlevel variables, *n* and ε, to the time derivatives of the macrolevel state variables, *S*, *N*, *E*, we cannot simply replace *n* with *N* and ε with *E* in the transition functions because of Jensen’s inequality and the nonlinearity of these functions. Instead, we average the transition functions over the dynamic structure function. That function, in turn, is determined in an iterative procedure, from the constraints imposed by the perturbed state variables and their time derivatives using MaxEnt. Figure 1 illustrates the recursive architecture of DynaMETE.

Importantly, our distinction between “steady‐state” and “dynamic” applies to the state variables only; in steady state, individuals can be growing or dying and abundances of species can be increasing or decreasing at any moment in time, provided the state variables are constant.

At any time, the constraints on the dynamic structure function, *R*, include the state variables and their first time derivatives. Two Lagrange multipliers, $\lambda_{1}$ and $\lambda_{2}$, correspond to the constraints provided by ratios of state variables, *N*/*S* and *E*/*S*, as in METE. Three additional Lagrange multipliers, $\lambda_{3},\;\lambda_{4}\;\mathrm{and}\;\lambda_{5},$ correspond to the constraints of (1/*S*)d*N*/d*t*, (1/*S*)d*E*/d*t* and d*S*/d*t*.

Consider an ecosystem that has been in steady state up until time *t* = 0; the state variables have been constant and the structure function is given by eqn 3. For *S*, those processes might include extinction, speciation and immigration; for *N*, they might include birth, death and immigration and for *E*, they might include ontogenic growth, death of individuals and immigration. At *t* = 0, a disturbance is imposed, perhaps a reduction in the immigration rate because of habitat fragmentation, or a change in the ontogenic growth rate or the per capita death rate because of climate change.

DynaMETE describes the time evolution of the system in the aftermath of that disturbance as an iterated sequence of steps (A‐G) in Fig. 1 and written more explicitly in Table 2.

**Table 2:** Inference dynamics. An iterative process for solving the dynamic state variable equations. Time is labelled with the subscript *i*. The notation {*X_i_*} is shorthand for the set of state variables, *S_i_*, *N_i_* and *E_i_*; {d*X_i_*/d*t*} is shorthand for the set of their time derivatives; and {*k*({X*_i_*}, {*c*_i_})} is shorthand for the set of dynamic transition functions, *f*, *h* and *q*

Step	Known	Derived from known
Before the perturbation		
1. In the steady‐state past, the static structure function is derived from static state variables; this step generates eqn 3.	$X_{\text{past}};\left\{\frac{dX_{\text{past}}}{dt}\right\}=0;$ $\lambda_{j,\mathrm{past}}=0\left(j=3,4,5\right)$	$\lambda_{1,\mathrm{past}};\;\;\lambda_{2,\mathrm{past}};\;R_{\mathrm{past}}$
Initialising the system right after the perturbation is imposed at t = 0		
2. A perturbation is now imposed, expressed by a change in one or more of the parameters, {*c*}, in the transition functions, *k*({*X*},{*c*}). The initial time derivatives of the state variables are calculated from eqns (16), (17), (18).	$\left\{X_{0}\right\}=\left\{X_{\mathrm{past}}\right\};\;{\lambda_{j,0}=\lambda }_{j,\mathrm{past}};$ *R*_0_ = *R*_past_; perturbed transition functions	$\{\frac{dX_{0}}{dt}\}\neq$ 0
After the system is initialised to the Perturbation		
3. The state variables are updated using their time derivatives using eqn 20.	$\left\{X_{0}\right\};\;\left\{\frac{dX_{0}}{dt}\right\}$	$\left\{X_{1}\right\}$
4. The transition functions are updated by substituting updated state variables.	{*k*({*X*_0_},{c_0_})}	{*k*({*X*_1_},{c_1_})}
5. The time derivatives of the state variables are updated using eqns (16), (17), (18).	{k({*X*_1_},{c_1_})};*R*_0_	$\left\{\frac{dX_{1}}{dt}\right\}$
6. The structure function is updated from the constraints derived above using eqns (9), (10), (11), (12), (13).	$\left\{X_{1}\right\};\left\{\frac{dX_{1}}{\mathrm{dt}}\right\}$; {*k*({*X*_1_},{c_1_})}	${\lambda_{1,1},\;..,\lambda_{5,1},\;R}_{1}$
Subsequent steps repeat steps 3–6. With each update of the structure function, the updated effects of the perturbation on abundance and metabolic rate distributions can be derived.		

To express Fig. 1 and Table 2 in equation form, we first introduce the transition functions. We denote the rate of change of the population of an arbitrary species by:

(11) dn/dt=f(n,ε,{X},{c})

Here, {*X*} refers to the set of state variables and {*c*} refers to the set of parameters such as migration rate or per capita birth and death rates that govern changes in the abundances of the species. We assume that *f* and the other transition functions do not depend on the {d*X*/d*t*}.

The metabolic rate of a species can change because of either a change in population size or because of ontogenic growth of an individual in the population. We denote the rate of change of the metabolic rate of an individual as:

(12) dε/dt=g(n,ε,{X}{c}),

Then

(13) $$\frac{d\left(\text{metabolic rate of a species}\right)}{\mathrm{dt}}=h\left(n,\varepsilon ,\left\{X\right\},\left\{c\right\}\right).$$

where the function *h* is constructed from *f* and *g* (see SI‐C). The functions *f* and *h* multiplied by *S* and averaged over the structure function give the time derivatives of *N* and *E* respectively (eqns 16 and 17 below).

Finally, the transition function $q\left(n,\varepsilon ,\left\{X\right\},\left\{c\right\}\right)$ describes processes governing changes in species richness, including extinction, immigration and speciation; averaged over the structure function it determines dS/dt (eqn 18 below).

To describe the iterative process, we introduce a discrete time‐step index, *i*, and designate the time‐dependent state variables, their time derivatives, the Lagrange multipliers, the transition rate parameters and the structure function as {*X_i_*}, {d*X_i_*/d*t*}, $\lambda_{j,i},$ {*c_i_*} and *R_i_* respectively. The index, *j,* designating the Lagrange multipliers, runs from 1 to 5.

Again consider a system that in the past (*i* < 0) was in steady state, with static state variables, static structure function given by eqn 3, and Lagrange multipliers given by eqns 5 and 6. For *i* < 0, $\lambda_{3,i},\;\lambda_{4,i},\;\lambda_{5,i}$ and the time derivatives of the state variables vanish. At *i* = 0, a disturbance is imposed that is expressible as a change in one or more of the parameters, *c*, in the transition functions. It can be a time‐varying or a fixed, one‐time parameter change.

The iterative process cycles through three groups of equations. First, at any moment in time, *i*, the structure function is calculated using MaxEnt with the constraints arising from the instantaneous values of the state variables and their time derivatives:

(14) $$N_{i}=S_{i}\sum_{n,\varepsilon }nR_{i}\left(n,\varepsilon ,\left\{X_{i}\right\},\;\left\{c_{i}\right\}\right)$$

(15) $$E_{i}=S_{i}\sum_{n,\varepsilon }n\varepsilon R_{i}\left(n,\varepsilon ,\left\{X_{i}\right\},\;\left\{c_{i}\right\}\right)$$

(16) $$\frac{dN_{i}}{\mathrm{dt}}=S_{i}\sum_{n,\varepsilon }f\left(n,\varepsilon ,{\{X}_{i}\},\left\{c_{i}\right\}\right)R_{i}\left(n,\varepsilon ,\left\{X_{i}\right\},\;\left\{c_{i}\right\}\right)$$

(17) $$\frac{dE_{i}}{\mathrm{dt}}=S_{i}\sum_{n,\varepsilon }h\left(n,\varepsilon ,{\{X}_{i}\},\left\{c_{i}\right\}\right)R_{i}\left(n,\varepsilon ,\left\{X_{i}\right\},\;\left\{c_{i}\right\}\right)$$

(18) $$\frac{dS_{i}}{\mathrm{dt}}=\sum_{n,\varepsilon }q\left(n,\varepsilon ,{\{X}_{i}\},\left\{c_{i}\right\}\right)R_{i}\left(n,\varepsilon ,\left\{X_{i}\right\},\;\left\{c_{i}\right\}\right)$$

Equations 14 and 15 impose the same constraints as do eqns 1 and 2; eqns 16 and 18 impose new constraints. For notational simplicity, conditionality of the structure function on the time derivatives of the state variables is implicit in eqns (9), (10), (11), (12), (13), whereas we have made explicit the dependence of the structure function on the state variables. Equations 14 and 18 implement step E in Fig. 1, step 6 in Table 2. From these equations, application of the MaxEnt inference procedure results in the following ecological structure function:

(19) $$R_{i}\left(n,\varepsilon ,\left\{X_{i}\right\},\left\{c_{i}\right\}\right)={Z_{i}^{‐1}e}^{‐\lambda_{1,i}n}e^{{‐\lambda }_{2,i}n\varepsilon }e^{{‐\lambda }_{3,i}f\left(n,\varepsilon ,\left\{X_{i}\right\},\left\{c_{i}\right\}\right)}e^{{‐\lambda }_{4,i}h\left(n,\varepsilon ,\left\{X_{i}\right\},\left\{c_{i}\right\}\right)}e^{{‐\lambda }_{5,i}q\left(n,\varepsilon ,\left\{X_{i}\right\},\left\{c_{i}\right\}\right)}$$

where *Z_i_* is a normalisation factor that depends on the $\lambda_{j,i}.$

To iterate the structure function, we need to update the state variables:

(20) $${\{X}_{i+1}\}={\{X}_{i}\}+\left\{\frac{dX_{i}}{dt}\right\}\Delta t\Delta t=1\;\mathrm{with}\;\mathrm{integer}\;\mathrm{index}\;i)$$

This is step B in Fig. 1, step 3 in Table 2. Equation 20 then allows us to directly update the transition functions by substitution (step C in Fig. 1, step 4 in Table 2).

Finally, we update the time derivatives of the state variables from time step *i* to *i* + 1 by averaging the transition functions over the structure function, with Lagrange multipliers determined from eqns (9), (10), (11), (12), (13) fixed at time step *i*, but the transition functions *f*, *h*, *q* appearing in the exponents of eqn 19 evaluated at the {*X_i_*_+1_}:

(21) $$S_{i+1}\sum_{n,\varepsilon }f\left(n,\varepsilon ,{\{X}_{i+1}\right\},\;\left\{c_{i+1}\right\})R_{i}\left(n,\varepsilon ,\;\left\{X_{i+1}\right\},\;\left\{c_{i+1}\right\}\right)=\frac{dN_{i+1}}{\mathrm{dt}}$$

(22) $$S_{i+1}\sum_{n,\varepsilon }h\left(n,\varepsilon ,{\{X}_{i+1}\right\},\left\{c_{i+1}\right\})R_{i}\left(n,\varepsilon ,\left\{X_{i+1}\right\},\;\left\{c_{i+1}\right\}\right)=\frac{\;dE_{i+1}}{\mathrm{dt}}$$

(23) $$\sum_{n,e\;}q\left(n,\varepsilon ,{\{X}_{i+1}\right\},\left\{c_{i+1}\right\})R_{i}\left(n,\varepsilon ,\left\{X_{i+1}\right\},\;\left\{c_{i+1}\right\}\right)=\frac{dS_{i+1}}{\mathrm{dt}}$$

The subscript *i* on $R_{i}$ in eqns (16), (17), (18) signifies that the Lagrange multipliers are those at step *i*, and thus depend on {*X_i_*} and {d*X_i_*/dt}. Equations 21 and 23 implement step D in Fig. 1, step 5 in Table 2.

Equations 14 and 23 comprise DynaMETE. They can be iterated to calculate the time evolution of both the state variables and the structure function. From the latter, the time‐dependent SAD and MRDI can be calculated (step F in Fig. 1).

We note that there is no mathematical or *de facto* connection between information entropy maximisation and equilibrium; at the completion of each entropy‐maximisation step, *S*, *N* and *E* will generally have non‐zero time derivatives, at least under disturbance, and hence the necessity, in subsequent iterations, to update their values, then update their time derivatives and then update the Lagrange multipliers. This is repeated until the system does, potentially, reach an equilibrium.

There are two distinct time scales in DynaMETE. Rate constants in the transition functions will have units of inverse time and for practical purposes this time scale might be conveniently described in units of years. Another time scale is the interval between updates in the iteration steps. In general, numerical accuracy will increase with more rapid updating; finding optimal time intervals for the iteration process remains to be explored.

Supporting Information‐A (SI‐A) explains the rationale for the term, *S*, multiplying the summations in eqns 16, 17, 21 and 22; SI‐B elaborates on the full set of eqns (9), (10), (11), (12), (13), (14), (15), (16), (17), (18), demonstrating their internal consistency.

We turn next to the model‐dependent, mechanistic parent of the hybrid theory: expressions for the transition functions *f*, *h* and *q*.

### The transition functions

Up until this point, we have described a very general theoretical framework that in principle could be applied to a wide variety of dynamical systems. To proceed, we make specific model assumptions about the mechanistic transition functions. There is no agreed‐upon set of processes governing the rates of change of *n* and ε, nor agreed‐upon mathematical representations of selected processes. As with all mechanistic modeling in ecology, plausible mathematical representations of very complex and imperfectly understood processes will necessarily be simplifications.

Our choices are guided by conformity with results from metabolic theory (Brown *et al*. 2004) and by scale‐consistency requirements as described below. The reasoning behind, and the derivations of, our choices of the explicit functional forms for the transition functions are given in SI‐C with some additional mathematical details about diversification via immigration in SI‐D. Alternative forms for the transition functions can readily be substituted for the ones selected here.

#### Summary of transition functions

From SI‐C we have

(24) $$f\left(n,\varepsilon \right)={{(b}_{0}‐d}_{0}E/E_{c})\frac{n}{\varepsilon^{1/3}}+m_{0}\frac{n}{N}$$

(25) $$h\left(n,\varepsilon \right)=w_{0}n\varepsilon^{2/3}‐\frac{w_{10}}{\ln^{2/3}\left(1/\beta \right)}n\varepsilon ‐d_{0}n\varepsilon^{2/3}E/E_{c}+\frac{m_{0}n}{N},$$

(26) $$q\left(n,\varepsilon \right)=\mu_{0}e^{‐\mu S‐\gamma }+\frac{\sigma_{1}KS}{K+S}+\frac{\sigma_{2}b_{0}nS}{\varepsilon^{1/3}}‐S\delta_{n,1}\frac{d_{0}E/E_{c}}{\varepsilon^{1/3}}.$$

All symbols in these equations are in the Glossary (Table 1). In eqn 24, birth and death rates are assumed to be proportional to *n*/$\varepsilon^{1/3}$ in conformity with metabolic scaling theory (West *et al*. 2001; Brown *et al*. 2004; Niklas 2007; Marba *et al*. 2007). The term *E*/*E_c_* represents a zero‐sum constraint that operates at community, not species or individual, level. In particular, *E_c_* is a metabolic rate limit for the community. Finally, it is assumed that if an immigrant is in a species already present in the local community, then the probability that it is in a species with *n* individuals in that community is just *n*/*N*; if the total immigration rate is *m*_0_, and the overwhelming fraction of immigrants is in existing species, then *m*_0_ *n*/*N* is the expected rate of immigration to a species with *n* individuals.

In eqn 25, the expression for ontogenic growth assumes metabolic scaling (West *et al*. 2001). The zero‐sum constraint and the contribution from immigration are based on the same assumptions as in eqn 24, and it is assumed that immigrants have ε=1. The term 1/ln^(2/3)^ (1/β) in the second term on the right‐hand side of eqn 25 is a scaling factor (see SI‐C).

In eqn 26, two different speciation models and immigration are included for generality. The expression multiplied by $\sigma_{1}$ corresponds to a speciation rate that for low species richness is proportional to the number of species in the community (Kirchner and Weil, 1998; Rabosky 2013) and saturates at high species richness. The term multiplied by $\sigma_{2}$ describes a speciation rate for the case that each birth has an equal chance of resulting in a new species (Weiser *et al*. 2018). Alternative functional forms of the speciation rate (e.g. Etienne & Rosindell 2012) could be used. The expression for the contribution to *q* from immigration in eqn 26 assumes that new species arriving in the local community originate from the relatively rare species in the metacommunity; a full derivation is given in SI‐D. The last term in eqn 26 describes extinction under the assumption that extinction occurs when the last remaining individual in a species in the local community dies.

With this tentative set of transition functions, a model realisation of the theory is completely specified. By iteration the time trajectories of the state variables, the structure function and the metrics of macroecology that derive from the structure function can be calculated.

## RESULTS

First, to explore DynaMETE at and near the steady state, we truncate the full iteration procedure at step 2 of Table 2 and derive a set of coupled time‐differential equations of motion (eqns (22), (23), (24), (25), (26), (27), (28), (29), (30), (31), (32)) that predict relationships among the state variables in steady state and the dependence of state variable trajectories on small perturbations in the parameters in the transition functions. These results should only be good approximations for small deviations from steady state because they derive from the static structure function (eqn 3) with $\lambda_{3},\;\lambda_{4},\;\lambda_{5}=0$.

Then, in a first iteration of the full theory (steps 3–6 in Table 2), we use eqns (9), (10), (11), (12), (13), (14), (15), (16), (17), (18) to calculate lowest order effects of various perturbations in the transition function rate constants on the structure function. From the perturbed structure function we then derive altered shapes of the abundance and metabolic rate distributions. We emphasise that these results may differ considerably from those obtained with higher order iterations.

### Predicted properties of State Variables at and Near Steady State

Using eqns (16), (17), (18), with transition functions specified in eqns (19), (20), (21) and *R* given by eqn 3, we obtain (see SI‐E for derivations):

(27) $$\frac{\mathrm{dN}}{\mathrm{dt}}=\;\left[b_{0}‐d_{0}\left(E/E_{c}\right)\right]\left[\frac{1.21N^{4/3}\ln^{1/3}\left(1/\beta \right)\left(1+\frac{4N\;\ln\;(1/\beta )}{3E}\right)}{E^{1/3}}‐\frac{3N^{2}\ln\left(1/\beta \right)}{2E}\right]+m_{0}$$

and

(28) $$\frac{\mathrm{dE}}{\mathrm{dt}}={[w}_{0}‐d_{0}\left(E/E_{c}\right)]\left[\frac{2.42E^{2/3}N^{1/3}}{\ln^{2/3}\left(1/\beta \right)}‐\frac{2.26E^{2/3}S^{1/3}}{\ln\left(1/\beta \right)}\right]\;‐\frac{w_{10}E}{\ln^{\frac{2}{3}}\left(1/\beta \right)}+\;m_{0}.$$

For the general case, with immigration and both speciation mechanisms operating, along with extinction, we have.

(29) $$\frac{\mathrm{dS}}{\mathrm{dt}}=m_{0}e^{‐\mu S‐\gamma }+\sigma_{1}\frac{\mathrm{KS}}{K+S}+\sigma_{2}b_{0}\left(\frac{1.21N^{4/3}\ln^{1/3}\left(1/\beta \right)}{E^{1/3}}\left(1+\frac{4N\ln\left(1/\beta \right)}{3E}\right)‐\frac{1.5N^{2}\ln\left(1/\beta \right)}{E}\right)‐\frac{{1.35\;d}_{0}}{E_{c}}\frac{S^{4/3}E^{2/3}}{\ln(1/\beta )}.$$

#### Steady states

Setting the time derivatives in eqns (22), (23), (24), (25), (26), (27), (28), (29), (30), (31), (32) to zero, we obtain the following relationships among the static values of the state variables and the parameters that describe the dynamics. From eqn 27:

(30) $$E=E_{c}\frac{b_{0}}{d_{0}}\left(1+\delta_{E}\right)$$

and from eqn 28:

(31) $$N={\left[\frac{0.41w_{10}}{w_{0}‐d_{0}\left(E/E_{c}\right)}\right]}^{3}E\left(1‐\delta_{N}\right).$$

The correction terms, $\delta_{E}$ and $\delta_{N},$ are of order ${(m}_{0}/b_{0})\left(\frac{E^{1/3}}{N^{4/3}}\right)$ and ${\left(S/E\right)}^{1/3}$ respectively, which will generally be << 1.

From eqn 29, for the immigration‐only case ($\sigma_{1}=\;\sigma_{2}$ = 0), we have

(32) $$Se^{3\mu S/4}={\left[\frac{0.41m_{0}\ln\left(1/\beta \right)}{d_{0}\left(E/E_{c}\right)}\right]}^{3/4}E^{1/4}$$

For the case $m_{0}=\;\sigma_{2}=0,$ and *S* >> *K*,

(33) $$S={\left[\frac{0.35\sigma_{1}\ln\left(1/\beta \right)}{d_{0}\left(E/E_{c}\right)}\right]}^{3/4}E^{1/4}$$

If *S* << *K*, then

(34) $$S={\left[\frac{0.35\sigma_{0}\ln\left(1/\beta \right)}{d_{0}\left(E/E_{c}\right)}\right]}^{3}E$$

Finally, for the case $m_{0}=\sigma_{1}=0:$

(35) $$S={\left[\frac{0.9\sigma_{2}b_{0}}{d_{0}\left(E/E_{c}\right)}\right]}^{3/4}\ln\left(1/\beta \right)E$$

#### The Steady‐State Species–Area Relationship (SAR)

We can derive nested SARs from eqns (26), (27), (28), (29) because in a nested design, *E_c_*, *E* and *N* scale linearly with area. In the immigration‐only model, taking the logarithm of eqn 32 gives:

(36) $$S=\frac{\ln(E)}{3\mu }+\frac{\ln(m_{0})}{\mu }‐\frac{4\ln(S)}{3\mu }+\frac{\ln(\ln(1/\beta ))}{\mu }+\frac{\ln\left(\frac{0.41E_{c}}{d_{0}E}\right)}{\mu }.$$

In a nested SAR design, *E_c_*, *E* and *N* scale linearly with area, and in general, *E* >> *m*_0_. Hence, *S* scales logarithmically with area, with ln(ln(area)) corrections arise from the third and fourth terms (see eqn 6) on the right‐hand side of eqn 36 and the fifth term contributes a constant. If *m*_0_ scales as a power of area, then the second term also gives a ln(area), which is generally smaller than the first term.

For sufficiently small *S* in eqn 32, the exponential term can be ignored, and then the first term on the right‐hand side of eqn 36 can be set equal to the third term. This results in *S* ~ $m_{0}^{3/4}E_{0}^{1/4}\;∼\;\mathrm{area}$ if *m*_0_ scales linearly with area, and *S* ~ area^1/4^ if *m*_0_ is scale independent. This limit applies when *S* < 1/μ, which for a typical 50 ha tropical forest plot is approximately *S* < 50 (see SI‐F).

**Figure 2:**
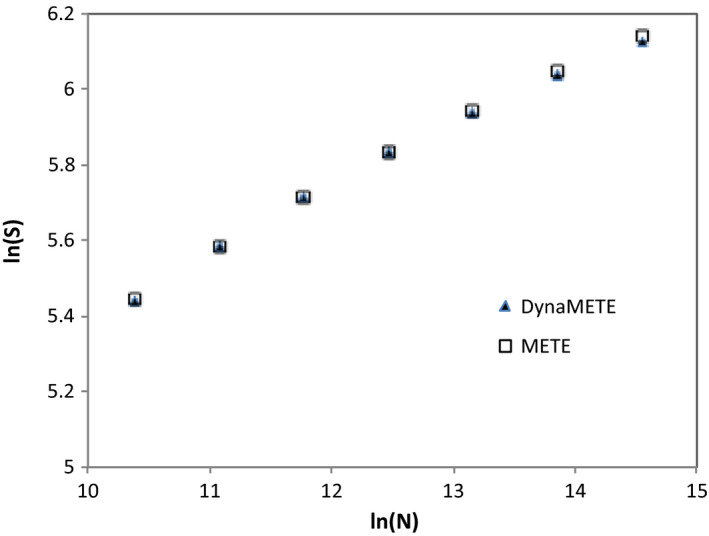
Comparison of up‐ and down‐scaled species richness using METE and DynaMETE, starting with identical species richness and abundance at the middle scale shown. The values of *N*, a proxy for area, span a scale range of 2^7^. Transition function parameters and *S* and *N* values for the middle scale are from Table 3; at larger or smaller scales *E*_c_ and *m*_0_ are assumed to scale linearly with area and the other parameters are held constant. We note that the SARs derived from METE using differing methods (McGlinn *et al*.^2013^) differ inconsequentially for the case of the BCI state variables and the scale range analysed here.

The METE SAR was derived (Harte 2011, eqn 7.68) from the predicted species‐ abundance and species‐level spatial‐occupancy distributions. The DynaMETE prediction does not depend on a spatial distribution function, but it does depend on the choices made for the mechanisms that govern the transition functions *f*, *h* and *q* which led to eqn 30. Nevertheless, METE and the static limit of DynaMETE in the migration‐only model predict similar functional forms for the SAR: *S* ~ ln(area) with ln(ln(area)) corrections. Numerical simulations for a variety of choices of state variables show that, beyond this functional similarity, they predict nearly overlapping SARs (see e.g. Fig. 2).

In the first speciation model, from eqn 33 with *S* >> *K* we get quarter‐power scaling of species richness with *E* (up to a $\ln\left(1/\beta \right)$ correction) and therefore with area. In that model, with *S* << *K*, and for any *S* in the second speciation model, *S* scales linearly with area, up to a ~ $\ln\left(1/\beta \right)$ln(*N*)‐ln(*S*) correction. The first model, with a saturation term *K* that is small compared to the steady‐state species richness, thus yields a more realistic species–area relationship.

#### A Productivity–biomass–diversity–abundance relationship at steady state

Metabolic scaling informs us that individual mass (*m*) is related to individual metabolism by $m\left(\varepsilon \right)=\;m\left(1\right)\varepsilon^{4/3}$. To scale this expression up from individual mass, *m*, to total community biomass, *B*, we again have to sum over the structure function. As derived in SI‐E, total biomass, *B*, is then:

(37) $$B=m\left(1\right)S\sum_{n,\varepsilon }\varepsilon^{4/3}nR(n,\varepsilon \left|S,N,E\right)=\;m\left(1\right)\frac{4.17E^{4/3}}{S^{1/3}\ln\left(1/\beta \right)}.$$

This community mass–metabolism relationship thus involves species richness, and also total abundance via the ln(1/β) term. Interpreting the state variable *E* as total net productivity *P* of the community, eqn 37 yields a relationship among productivity, biomass, species richness and abundance:

(38) $${{P=0.343\;B}^{3/4}S}^{1/4}\ln^{3/4}\left(1/\beta \right)$$

where *B* and *P* are measured in units such that m(ε=1)=1. Equation 38 does not depend on whether migration, speciation or a combination of both contributes to diversification because eqn 29 was not used in its derivation. Nor does it depend on the forms of the transition functions and the rate constants, which will differ from habitat to habitat.

Noting the different scaling exponents in the contribution of biomass and species richness to *P*, and that ln(1/β) varies approximately as ln(*N*) – ln(*S*), the influence of biomass on productivity is considerably stronger than that of species richness, which in turn is stronger than that of abundance, which only enters via the ln(1/β) term. Moreover, with productivity and abundance fixed, then up to a logarithmic correction, total biomass varies as *S* ^‐1/3^. Empirical surveys of the productivity**–**biomass**–**richness**–**abundance relationship (Ghedini *et al*. 2018; Jenkins 2015; Niklas 2007) are qualitatively consistent with these results but extensive analyses will be required to test eqn 38.

Equation 38 is an “ideal biodiversity law”, an analog of the ideal gas law that relates thermodynamic state variables. Because this equation was derived using the steady‐state structure function in eqn 3, it will likely no longer hold when the state variables are trending up or down. Following disturbance, the full structure function (eqn 19) is needed to derive the productivity**–**biomass**–**abundance**–**species richness relationship, and it will then depend on the details of the disturbance mechanism.

We turn now from the static limit of DynaMETE to its dynamic predictions, examining both state variable dynamics and the shapes of the SAD and MRDI away from steady state.

#### State variable dynamics near steady state

Time trajectories of the state variables near steady state follow from eqns (22), (23), (24), (25), (26), (27), (28), (29), (30), (31), (32), which were derived from the static structure function. We examine both the first‐order responses of the state variables to several kinds of disturbance, expressed by altered transition rate parameters, and the recovery to steady state from a depauperate state.

For these dynamical simulations, we specify the transition rate parameters for a forest resembling the 50 ha BCI tropical forest plot (Condit *et al*. 2000; Condit, 1998; Condit et al. 2019; Hubbell *et al.,* 1999). Approximate transition rate constants for this site are given in Table 3 and the rationale for these values is given in SI‐F.

**Table 3:** Parameter values for the transition functions and state variables in a BCI‐like forest ecosystem in which diversification is driven solely by immigration. The rationale for the parameter values is given in SI‐F

Parameter	Value
*b*_0_	0.2
*d*_0_	0.2
*m*_0_	500
*w*_0_	1.0
*w*_10_	0.4096
*E*_c_	2 × 10^7^
$\mu =S_{\mathrm{meta}}^{‐1}\ln\left(1/\beta_{\mathrm{meta}}\right)$	0.0219
State variables	
*S*	320
*N*	230 000
*E*	2.04 × 10^7^

**Figure 3:**
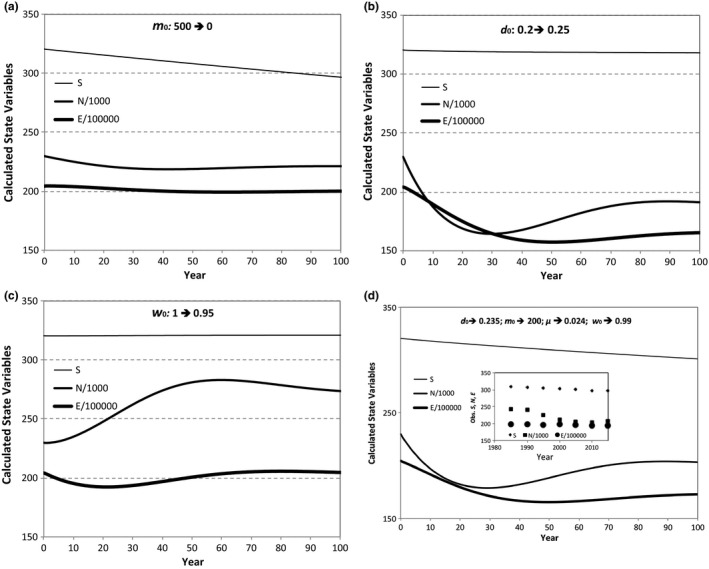
Responses of state variables to perturbations simulated from Eqns (22), (23), (24), (25), (26), (27), (28), (29), (30), (31), (32): a. reduction of immigration rate, *m*_0_; b. increase in death rate, *d*_0_; c. reduction in growth rate, $\omega_{0};$ d. increase in the death rate and reduction in the immigration and ontogenic growth rates. The inset in 3d shows the state variable trajectories from 1985 to 2015 in the BCI 50 ha tropical forest plot. The censuses include trees with dbh ≥ 1 cm. Data from Condit (2019); Hubbell *et al*. (2005). The inset assumes that the metabolic rate of individuals scales linearly with basal area.

Figures 3a–d illustrate responses of the state variables, over 100 years, to different disturbances represented by changing the values of the rate parameters in the transition functions. Independent of the magnitude of the changes in these parameters, certain general patterns emerge. A decrease in the immigration rate constant, for example, under habitat fragmentation that isolates an ecosystem from its metacommunity, results in a linear decrease in *S* at small *t*, as well as a weak, damped oscillatory response of *N*, and nearly undetectable change in *E* (Fig. 3a). An increase in the death rate (Fig. 3b) generates a slight decrease in *S*, an initial large decline in *N* and a weaker decline in *E*, followed by damped oscillatory behavior. A decrease in the ontogenic growth rate (Fig. 3c) generates a nearly indiscernible increase in *S*, a large damped oscillatory initial rise in *N* and weak damped oscillatory initial decrease in *E*.

Figure 3d shows the effect on state variable trajectories of combining perturbations in migration, death and growth rates. We do not attempt here a detailed comparison with real data because of the first‐order approximation used to obtain these theoretical curves, but it is encouraging that the time trajectories of the BCI state variables over the period 1985–2015 (inset in Fig. 3d) also exhibit a steady decline in *S*, decline and then partial recovery in *N* and weak variability in *E*.

**Figure 4:**
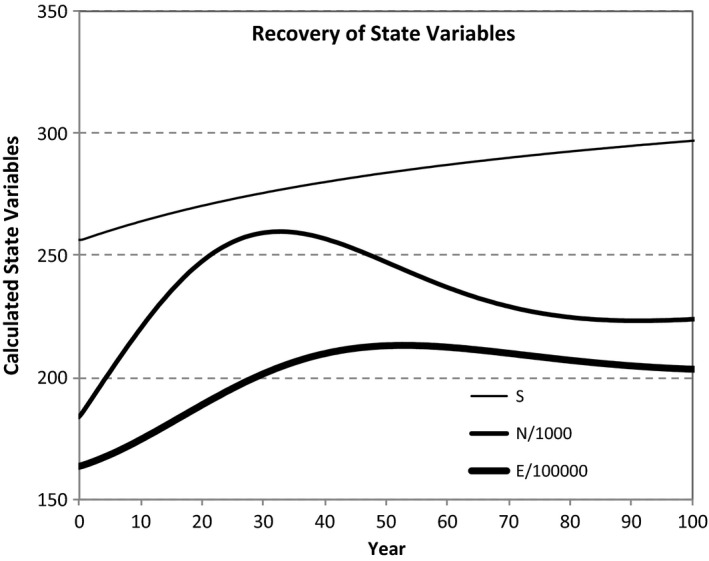
Predicted recovery of state variables to their steady‐state values in Table 3, as predicted from Eqns (22), (23), (24), (25), (26), (27), (28), (29), (30), (31), (32), with steady‐state parameters and each initial state variable equal to 80% of its steady‐state value. The monotonic rise in S to steady state, along with the sizeable overshoot and then damped oscillation in *N*, and the smaller overshoot and then damped oscillation in *E* occur for a wide range of initially depleted state variables, steady‐state variables and parameter choices.

If we fix the transition rate parameters at their undisturbed values (Table 3) in the immigration‐only model, and initially reduce the state variables 20% from their steady‐state values, their return to steady state is shown in Fig. 4. Noteworthy is the monotonic recovery of *S on a time scale of centuries*, the large overshoot and then decline to steady state of *N*, and a much weaker overshoot and decline of *E*.

If speciation is the driver of diversification, the pattern of recovery of species richness is markedly different. In the first speciation model, with *K* >> *S*, recovery of *S* is sigmoidal and extremely slow, with 90% recovery taking approximately 10^4^ years. In the second speciation model, and in the first with *K* << *S*, *S* recovers to 90% of steady state in approximately 4000 years, and at an ever‐slowing, rather than sigmoidal, rate. The recovery trajectories of *N* and *E* are nearly the same in both speciation models and very similar to that in the immigration‐only case (Fig. 4).

### Perturbed abundance and metabolic rate distributions in DynaMETE

Here, we examine, in a first approximation to a fully iterated solution, how different types of disturbance give rise to characteristic departures of the SAD, $\phi \left(n\right),$ and the MRDI, $\psi \left(\varepsilon \right),$ from their steady‐state form. In particular, we truncate the iteration procedure, stopping with step 6 in Table 2. A single iteration at a one‐year time step, however, results in changes in the structure function that are too small to show interesting deviations from steady state, so to generate a discernible effect for a single iteration we use a time step of 25 years. Specifically, we assume the static structure function, with Lagrange multipliers “frozen” at their static numerical values as prescribed in eqns (16), (17), (18), perturb the transition functions by changing one or more rates constants and then derive from eqns (16), (17), (18) a set of time‐differential equations for the state variables. These equations differ from eqns (22), (23), (24), (25), (26), (27), (28), (29), (30), (31), (32) because the latter were derived by updating the Lagrange multipliers at each time step. We then ran these equations out to *t* = 25 and take the values of the {*X*(25)} and the {d*X*(25)/dt} as constraints in eqns (9), (10), (11), (12), (13) to calculate using MaxEnt a perturbed structure function. That function will be of the form of eqn 19 and from it we derive perturbed forms for the species abundance distribution (SAD) and the metabolic rate distribution over individuals (MRDI) using the same summations as performed to derive eqns (6), (7).

**Figure 5:**
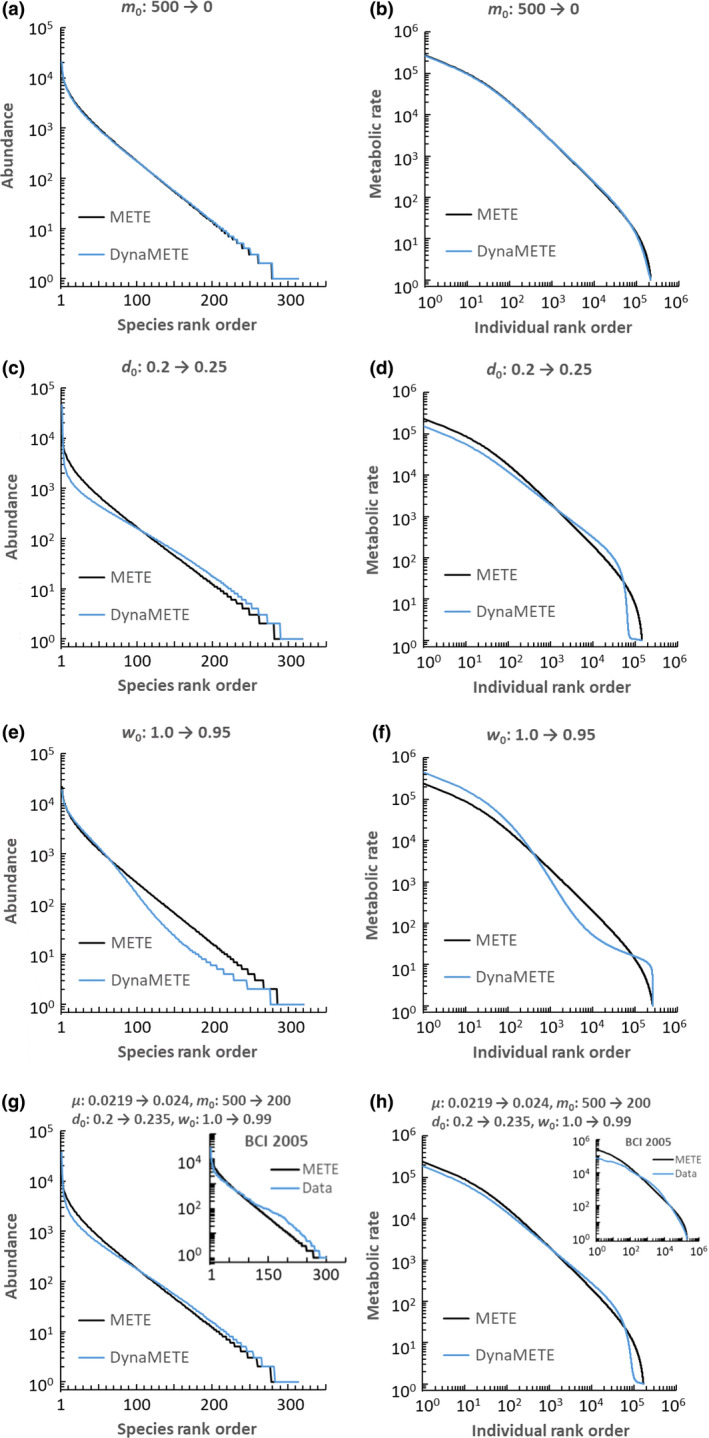
The effect of perturbations on the species abundance distribution (SAD) and the distribution of metabolic rates over individuals (MRDI). The relevant perturbation is in each plot’s title. Insets in Figs 5g and h show BCI data (see caption to Fig. 3). Note that the overlapping lines at ln(*n*) = 0 for the METE and DynaMETE SAD predictions actually extend to the same maximum rank in all cases.

The results are shown in Fig. 5. The five derived Lagrange multipliers are given in Table 4. Setting the immigration rate constant, *m*_0_, to zero only slightly alters the SAD and the MRDI in this first iteration of the full structure function (Fig. 5a and b). A 25% increase in the death rate constant, *d*_0_, shifts the SAD towards a lognormal shape as indicated by the curved rank‐log(abundance) graph at intermediate abundances (Fig. 5c). The rank‐log(metabolism) graph shifts in a more complex manner, weaving around the METE prediction, and predicting more of the very smallest trees (ε=1), fewer individuals with low (ε=2‐100) metabolism, and more trees with relatively high (ε=100‐100 000) metabolism, and a reduction in the sizes of the very largest individuals (Fig. 5d). A 5% decrease in the growth rate of individuals, *w*_0_, generates a roughly mirror‐image shift in the SAD relative to that from an increase in the death rate; the resulting SAD is approximately described by either an exponential distribution or an inverse power function with exponent >1 (Fig. 5e). Similarly, the shift in the MRDI generated by a decrease in growth rate is roughly the mirror image of the shift induced by an increase in the death rate (Fig. 5f).

**Table 4:** Perturbations, constraints and resulting Lagrange Multipliers used to generate Figures 5a–h. The text describes how the constraints are derived

	*m*_0_ = 500→0	*d*_0_ = 0.2→0.25	*w*_0_ = 1.0→0.95	μ = 0.0219→0.024 *m*_0_ = 500→200 *d*_0_ = 0.2→0.235 *w*_0_ = 1.0→0.99
Figure	5a, 5b	5c, 5d	5e, 5f	5g, 5h
State variables and their rates of change used for constraints				
*S*	314.2	319.6	320.8	314.9
*N*	217 962	144 430	262 537	166 984
*E*	2.0380 × 10^7^	1.7331 × 10^7^	1.8280 × 10^7^	1.7822 × 10^7^
d*S/*d*t*	−0.242	−0.009	0.0246	−0.196
d*N/*d*t*	−418	−1052	2350	−758
d*E/*d*t*	−4349	−112262	−48162	−88709
Lagrange multipliers				
$\lambda_{1}$	0.00037020	0.0023398	−0.0073972	0.0014027
$\lambda_{2}$	0.000014717	9.6690 × 10^−6^	0.000054585	0.000012665
$\lambda_{3}$	0.074392	0.16739	0.58409	0.16072
$\lambda_{4}$	−0.000015435	−0.00019515	0.00050473	−0.00010897
$\lambda_{5}$	−0.0049918	−0.015078	−0.27931	−0.0091964

Figures 5g and h show the effect of the same combination of changes in the rate constants used to generate Fig. 3d. We do not attempt here a detailed comparison with real data because of the first‐order approximation used to obtain these theoretical curves, but note their rough similarity to the empirical SAD and MRDI at BCI (see insets in Figs 5g and h).

We emphasise that a full iterative solution of eqns (9), (10), (11), (12), (13), (14), (15), (16), (17), (18) in, say, 25 one‐year time steps, over a period of 25 years could result in output that differs from the truncated solutions in Fig. 5 because of nonlinearities in the transition functions. The results in Fig. 5 are like a first‐order term in a Taylor's series. Higher order iteration is needed to extend the predictions out farther in time.

## DISCUSSION

METE's failure to predict macroecological phenomena in disturbed, dynamic ecosystems is the motivation for DynaMETE. DynaMETE hybridises mechanism and MaxEnt, and as a result the theory's predictions deviate from METE in a way that depends upon the mechanism of disturbance.

Dynamics in DynaMETE are described by mechanisms governing the microscale level of individuals and populations within the constraints of metabolic scaling theory (Brown *et al*. 2004). Upscaling to the macrolevel of communities is accomplished by averaging the mechanistic transition functions over the structure function that derives from MaxEnt. This leads naturally to an iterative procedure summarised in Fig. 1 and Table 2 and written out explicitly in eqns (9), (10), (11), (12), (13), (14), (15), (16), (17), (18). Whether this approach, or alternatives such as use of master equations to derive stochastic realisations of neutral population models (Hubbell, 2001; O’Dwyer *et al.*, 2009; Overcast *et al.,* 2019) results in more accurate predictions remains to be evaluated.

### Summary of major predictions

#### Static limit

In its static limit, DynaMETE recovers the static METE predictions for the distributions of abundances over species and metabolic rates over individuals, but also makes new predictions. First, is a scaling relationship among productivity, biomass, species richness and abundance in steady state (eqn 38); this relationship remains to be tested. Second, in the version of DynaMETE in which only immigration, not speciation, contributes to diversification, DynaMETE predicts a static‐limit SAR increasing approximately as ln(*A*), with a ln(ln(*A*)) correction, in very close agreement with the static SAR predicted by METE (Fig. 2).

If speciation but not immigration is assumed to drive diversification, then the SAR predicted by DynaMETE depends strongly on the form of the dependence of the speciation rate on the variables *S*, *N*, *n* and ε. In particular, if the speciation rate is proportional to the birth rate (eqn 35), or is an unsaturated function of *S* (*S* << *K*; eqn 34) in steady state, then the SAR is approximately linear in area at all scales, contrary to multiple observations. In the saturation model, if steady state is achieved with *S* >> *K*, we obtain a more realistic SAR (eqn 33), but *S* >> *K* contradicts the original motivation for *S*‐dependent speciation. DynaMETE suggests a testable alternative: a logarithmic dependence of speciation on species richness. Using the methods in SI‐E, this results in the plausible SAR:

(39) $$\frac{S}{\ln^{3/4}\left(S\right)}∼\;{\left(A\right)}^{1/4}\ln^{3/4}\left(1/\beta \right)$$

and unsaturated dependence of diversification on S.

#### Dynamics

Assuming a static structure function (eqn 3) in a lowest order solution to the theory, eqns (22), (23), (24), (25), (26), (27), (28), (29), (30), (31), (32) predict signature time trajectories of the state variables for various types of perturbations in the transition function rate constants (Figs 3). A combination of reduced immigration and growth rates and increased death rate generates trajectories that resemble those observed in the BCI tropical forest plot (see inset in Fig. 3d). Because these model simulations are based on extrapolating out in time only the first‐order iteration of the full theory, they are only suggestive; empirical testing awaits further iteration of eqns (9), (10), (11), (12), (13), (14), (15), (16), (17), (18).

DynaMETE also predicts trajectories of state variables during the recovery of an ecosystem from a depauperate state. The predicted overshoot and then decline to steady state of *N* (Fig. 4) suggests the “dog hair” stage of forest succession in which, post fire or other disturbance, the abundance of small trees increases rapidly, *E*/*N* decreases and then self‐thinning brings the system to a quasi‐steady state, until the next disturbance.

DynaMETE also predicts that different types of perturbations result in different patterns of deviation from static METE predictions (Fig. 5) for both the distribution of abundances over species and metabolic rates over individuals. These signature patterns can provide a way to identify the processes that are driving ecosystem change under natural or anthropogenic disturbance.

The SAD in the 50‐ha BCI tropical forest plot, which deviates from the log‐series distribution predicted by METE, resembles the DynaMETE prediction assuming a combined perturbation in death, growth and migration rates. That same perturbation also results in an MRDI that improves on the METE prediction for the high‐metabolising (large size) individuals, but over‐predicts the number of the lowest metabolism individuals. Further study of different combinations of parameter perturbations coupled with analysis of higher order iterations of the dynamics is needed.

### Future work

#### Flexibility of transition functions

Our assumptions about the functional forms of the transition functions are replaced with alternatives. The extinction process (last term in eqn 26) can be modified to describe a minimum viable population size > 2. Alternative forms of the ontogenic growth equation (e.g. Makareiva *et al.,* 2004) can be substituted for the first term on the right‐hand side of eqn 25. Density dependence can be described by a –d_1_ *n* ^2^ term instead of by our community‐level energy constraint. Moreover, a wide range of options are possible for modeling the dependence of speciation rates on *n*, ε and the state variables. A modification of the birth rate function can improve the realism of the transition functions when applied to forest census data that are limited to trees with some minimum threshold dbh, as, for example, with the Smithsonian Tropical Forest census data. In such systems, entry into the data set arises not from birth but from ontogenic growth into the smallest censused size cohort.

#### Approaches to higher order iteration

Preliminary exploration of higher order iterations in DynaMETE suggests that the MaxEnt optimisation equations for the Lagrange multipliers can be time intensive to solve numerically. We are currently evaluating a novel analytic approach to deriving and solving simultaneous linear differential equations for the time derivatives of the Lagrange multipliers.

#### Space

To include space explicitly in DynaMETE, one could modify the assembly (or colonisation) rule (Harte, 2011) that generates the species‐level spatial distributions in METE (Conlisk *et al*. 2007; Brush & Harte 2021) by combining the assembly rule with explicit demographic perturbations incorporated in the transition function *f*(*n*, ε).

#### Traits

DynaMETE currently incorporates two traits: individual metabolic rate (or body size) and species abundance. Other traits are ignored or implicitly assumed to be neutrally distributed across species and individuals. METE predicts an inverse relationship, referred to as energy equivalence (Damuth 1981; Brown *et al*. 2004), between the abundance of a species, *n*, and the average metabolic rate of its individuals, <ε|n>. METE and DynaMETE can be extended to include additional variables corresponding to other properties or traits of individuals and species, as well as analogs of energy equivalence for these. For example, if one adds to the list of state variables a new macrolevel resource variable, *W* (for water availability), and an associated individual water uptake rate, *w*, then METE predicts a modified SAD of the form $\phi \left(n\right)∼\exp\left(‐\beta n\right)/n^{2}$ (Harte & Newman 2014), and thus more rarity than results from the log‐series distribution. If the transition functions depend upon the individual water use efficiencies, ε/*w*, then the effect of water scarcity and differing water use efficiency distributions on the SAD and the MRDI could be investigated.

#### Higher taxonomic categories

The scope and realism of static METE were previously enhanced by including an additional state variable corresponding to the number of families in the community (Harte *et al.,* 2015). DynaMETE can also be readily modified to include additional or alternative state variables corresponding to the richness of higher taxonomic categories or, using lineages in a phylogeny, the elapsed distance separating pairs of individuals from their nearest shared ancestor.

#### Some broader issues

Both anthropogenic stresses and natural disturbances can cause state variables to rapidly change, and thus systems experiencing either type of disturbance can fall within DynaMETE’s criterion for a dynamic ecosystem. Although evidence reviewed in the Introduction suggests that systems undergoing either type of disturbance exhibit macroecological patterns that deviate from METE, the applicability of DynaMETE both to anthropogenically disturbed ecosystems and to naturally disturbed systems is not known. Given the importance of finding early warning signals that distinguish human impact on ecosystems from the effects of natural disturbance regimes, this is a high priority.

Gaston & Blackburn (2000) argued that no fundamental new insight will be obtained by comparing patterns along disturbance gradients because the rules that govern how process determines pattern will be the same in all ecosystems. In the MaxEnt inference approach to macroecology, however, when the numerous mechanisms that influence ecosystems are in balance, resulting in static state variables, then patterns do not depend on process (Harte & Newman 2014). In disturbed systems, however, with dynamic state variables, DynaMETE asserts that the actual mechanism of disturbance now governs macroecological patterns. In other words, the rules have indeed changed under disturbance.

In a provocative article, Goldenfeld & Woese (2011) suggest that while physics makes a clean separation between the state of a physical system and the equations that govern the time evolution of the system, successful biological theory will inevitably be self‐referential or recursive in the sense that the state of the system will strongly enter into the equations that govern dynamics. We observe that this is true of DynaMETE (Eqns (9), (10), (11), (12), (13), (14), (15), (16), (17), (18); 24–26); the state variables appear explicitly in the transition functions, which in turn govern state dynamics.

Many academic fields seek to unify complex micro‐ and macrolevel dynamics, in a speculative vein we suggest that the proposed iterative procedure at the core of DynaMETE could be of possible application in, for example, economics (Golan, 2018) and in statistical physics (Jaynes, 1957, 1982), where in both of these fields, static equilibrial patterns can be captured by MaxEnt but non‐equilibrial dynamics has remained elusive.

## CONCLUSION

Although ecosystems are dynamic and macroecological patterns do not remain constant in the face of disturbance, dynamic macroecology has not been adequately explored. DynaMETE, a theory of dynamic macroecology, hybridises explicit mechanisms driving change with a powerful inference procedure, MaxEnt, from information theory. By predicting how patterns in macroecology shift under anthropogenic perturbations, or under natural successional and evolutionary forces, DynaMETE can contribute to better understanding the fate of disturbed ecosystems, to improving conservation and management strategies in ecology, to developing early warning indicators of ecosystems in transition or at the edge of collapse, to identifying specific processes driving ecological change and to clarifying the roles of ecology and evolution in diversifying ecosystems. DynaMETE is a candidate dynamic theory of macroecology in the Anthropocene.

## AUTHORSHIP

JH conceived DynaMETE and led the manuscript writing; MB and KU contributed to theory formulation, analysis and manuscript writing; KU carried out numerical simulations.

### Peer Review

The peer review history for this article is available at https://publons.com/publon/10.1111/ele.13714.

## Supporting information

Supplementary Material

## Data Availability

No new data were used. The BCI data can be found at https://forestgeo.si.edu/explore‐data and are available from the Dryad Digital Repository at https://doi.org/10.15146/5xcp‐0d46.
