# Supplementary material for: DynaMETE: a hybrid MaxEnt‐plus‐mechanism theory of dynamic macroecology
Source: Ecol Lett. 2021 Mar 6;24(5):935–49. doi: 10.1111/ele.13714 (PMC8251983; doi:10.1111/ele.13714)
Supplement: Supplementary file 1 — Supplementary Material [file ELE-24-935-s001.docx]

**Supporting Information for**

**DynaMETE: A Hybrid MaxEnt-Plus-Mechanism Theory of Dynamic Macroecology**

**John Harte, Micah Brush, Kaito Umemura**

| **CONTENTS** | **pg** |
| --- | --- |
| **SI-A. The dynamic constraint equations.** | **2** |
| **SI-B. Internal consistency of the iteration scheme (Eqs. 14-23 in text).** | **3** |
| **SI-C. Motivation for explicit forms of transition functions (Eqs. 24-26 in text)** | **5** |
| **SI-D. Derivation of the rate of diversification from immigration (first term on**  **right-hand side of Eq. 26 in text).** | **10** |
| **SI-E. Derivations of Eqs. 27-29; 37 in text.** | **12** |
| **SI-F. Justification for the parameter values in Table 3.** | **19** |

**SI-A. The dynamic constraint equations.**

Eqs. 16 and 17 require some explanation. It is not obvious, for example, whether the average of d*n*/d*t* over *R*_,_ is equal to (1/*S*)d*N*/d*t* or d(*N*/*S*)/d*t*, or perhaps something else. The potential ambiguity arises because a death event will lead to a decrease in *N* and, if there are singleton species present, it can lead to a decrease in *S*. To see why Eq. 16 is written the way it is, consider, first, the case in which the death rate is proportional to the number of individuals in a species, and let the initial number of species be *S* and the initial number of individuals be *N*. Further assume that of the S species, *j* of them have only a single individual.

Writing the initial list of abundances as $n_{1}, n_{2}, \ldots,n_{j}, n_{j+1},\ldots,n_{S}$, where *n*_1_, …,*n*_j_ = 1, a single death event will lead to the following outcomes, with specified probabilities. Each of the j singletons has a probability of 1/*N* of losing an individual and hence of the system losing a species. Each of the *S* – *j* non-singletons has a probability of *n*_i_/*N* of losing an individual without loss of a species. After a single death event, and averaging over all the choices for which species loses an individual, the average abundance of each of the first *j* species is 1-1/*N*, and the average abundance of each of the *S* – *j* species with more than one individual initially is n_i_(1-1/*N*), i = *j*+1, …, *S*. Hence the expected average change in the abundances of the species, the average of *n* is –[*j*/*N* + (*N*-*j*)/*N*]/*S* = -1/*S*. Moreover, the average number of species remaining after the single death event is *S* – j/*N* and so the expected change in the number of species is $\Delta S= -j/N$. Because the change in the total number of individuals, $\Delta N,$ is -1, the average of $\Delta n$ is equal to $\Delta N/S$ and not to $\Delta\left( N/S \right).$

The same reasoning also works under the alternative assumption that the probability of a death event is the same for all species or that it depends on any arbitrary function of the abundance of species such as *n*^2^. In all cases, the average of d*n*/d*t* is given by (1/*S*)d*N*/dt, as in Eq. 16. Similar reasoning shows that the expression on the left-hand side of Eq. 17 is also correct.

**SI-B. Internal consistency of the iteration scheme (Eqs. 14-23 in text).**

Our iteration scheme is novel and therefore a consistency check is warranted. To simplify the argument, consider first the case in which the state variables and their time derivatives have no explicit time dependence (that is, their partial derivatives with respect to time vanish), which is equivalent to saying that the functions, *f*, *h*, *q*, describing the dynamics at the micro-level do not have explicit time dependence or, in other words, the rate constants appearing in those functions are true constants.

We begin with the simplified pair of equations that arise in a system with only one time-varying state variable, which we take to be *N*. Eqs. 16 and 21 then read:

$\frac{dN_{i}}{dt}=S\sum_{n,\varepsilon} f(n,\varepsilon,N_{i})R_{i}\left( n,\varepsilon,N_{i} \right)$ (B-1)

and

$\frac{dN_{i+1}}{dt}=S\sum_{n,\varepsilon} f(n,\varepsilon,N_{i+1})R_{i}\left( n,\varepsilon,N_{i+1} \right)$ (B-2)

where we have made explicit the dependence of *R* on the state variables as a consequence of the appearance of the transition functions, themselves dependent on the state variables, in the exponents in Eq. 19. Importantly, note that the structure function in both Eqs. B-1 and B-2 is the *t* = *i* function, which means that the Lagrange multipliers are numerically equal to those found from the constraints at *t* = *i*.

Subtracting Eq. B-1 from B-2, and taking the limit $\Delta t$ $\to\mathrm{dt}$, we have:

$\frac{d^{2}N_{i}}{d^{2}t}dt=S\sum_{n,\varepsilon} f(n,\varepsilon,N_{i+1})R_{i}(n,\varepsilon,N_{i+1})-S\sum_{n,\varepsilon} f(n,\varepsilon,N_{i})R_{i}(n,\varepsilon,N_{i})$ (B-3)

Using Eq. B-1, the right-hand side of B-3 is $\left( \frac{\Delta\left( \frac{dN}{dt} \right)}{\Delta N} \right)\Delta N,$ or in the continuous limit,

$\frac{d^{2}N_{i}}{d^{2}t} =\frac{\partial\frac{dN_{i}}{dt}}{\partial N_{i}}\frac{dN_{i}}{dt}$ (B-4)

Given our assumptions, this is an identity, establishing the internal consistency of our iterative scheme. In other words, we can derive Eq. B-2 from Eqs. B-1 and B-4.

If we had included all three state variables, thenwe would obtain:

$\frac{d^{2}N_{i}}{d^{2}t}=\frac{\partial\frac{dN_{i}}{dt}}{\partial N_{i}}\frac{dN_{i}}{dt}+\frac{\partial\frac{dN_{i}}{dt}}{\partial S_{i}}\frac{dS_{i}}{dt}+\frac{\partial\frac{dN_{i}}{dt}}{\partial E_{i}}\frac{dE_{i}}{dt}$ (B-5)

If the dynamics are, themselves, time dependent because one or more of the rate constants depend explicitly on time, then we would have to include partial derivatives with respect to time in the above derivation, but the end result would still be an identity.

**SI-C. Motivation for explicit forms of transition functions (Eqs. 24-26 in text).**

Consider, first, an arbitrarily chosen species with *n* individuals. The rate of change of *n* is assumed to depend upon the birth and death rate of its individuals and the migration rate of individuals into that species. The birth and death rates of an individual in the species will in general depend upon the metabolic rate of the individual, and hence the function *f*(*n*,$\varepsilon)$ = d*n*/d*t* will depend upon both *n* and $\varepsilon$. The species chosen in arbitrary in the sense that *S*^-1^d*N*/dt is given by the average of *f* over the structure function, *R*.

Similarly, *S*^-1^d*E*/d*t* is given by averaging a function *h*(*n*,$\varepsilon)$over the structure function, where *h*(*n*,$\varepsilon)$ = d(metabolic rate)/d*t* for an arbitrary species. Two kinds of terms will contribute to *h*: changes in the metabolic rate of an individual in the species as a result of ontogenic growth, which we denote by g($\varepsilon$) = d$\varepsilon$/d*t*, and changes in total species metabolism that arise because of changes in *n* resulting from birth, death and migration*.*

For notational simplicity, we suppress in this subsection the time index for the state variables and rate constants that the transition functions depend upon.

**Contribution of birth and death to *f*(*n*,**$\boldsymbol{\varepsilon)}$. Species-level demographics is constrained by metabolic scaling theory, so that per-capita birth and death rate scale inversely with the 1/3 power of the average metabolic rate of the individuals in the species (Niklas 2007; Marba et al. 2007):

$f_{\mathrm{birth}}=b_{0}\frac{n}{\varepsilon^{1/3}}$ (C-1)

Here, and in the other transitions that follow, a correlation between *n* and $\varepsilon$ in the form of an energy equivalence relationship (Brown et al., 2004) arises when expressions such as $n/{\varepsilon^{1/3}}$ are averaged over the structure function *R*(*n*, $\varepsilon$) (Harte et al., 2008).

The contribution of death to d*n*/d*t* also depends on $n/{\varepsilon^{1/3}}$but with a weak zero-sum constraint which operates at the community level and arises from the value of *E*, not *N* or *n*. Thus, we multiply $n/{\varepsilon^{1/3}}$by *E*/*E*_c_, where *E*_c_ is a soft metabolic limit:

$f_{death}=-\frac{d_{0}n}{\varepsilon^{1/3}}E/{E_{c}}$ (C-2)

When we examine the spatial scaling properties of DynaMETE, the parameter *E*_c_ will also serve as a fundamental scale parameter, with *E*_c_ proportional to plot area within a given habitat.

**Contribution of death to *h*(*n*,**$\boldsymbol{\varepsilon).}$Multiplying the death rate in Eq. C-2 by *ε*, we get the following contribution to *h*(*n*,$\varepsilon):$

$h_{\mathrm{death}}={-d}_{0}n\varepsilon^{2/3}E/{E_{c}}$ (C-3)

We ignore the contribution of birth to *dE/dt* because birth primarily partitions, rather than adds to, metabolism.

**Contribution of immigration to *f*(*n*,**$\boldsymbol{\varepsilon)}$ **and *h*(*n*,**$\boldsymbol{\varepsilon).}$ For a vegetation community, we denote by *m*_0_ the immigration rate of seeds that result in germinants with $\varepsilon$= 1. The probability that the immigrant is in an existing species with abundance *n* is assumed to be *n*/*N*, so:

$f_{\mathrm{immigration}}=m_{0}\frac{n}{N}$ (C-4)

Because the metabolic rate of these immigrants is 1:

$h_{\mathrm{immigration}}=m_{0}\frac{n}{N}$ . (C-5)

For an animal community, Eq.C-5 would be multiplied by the metabolic rate of individuals dispersing into the plot.

**Contribution of ontogenic growth to *h*(*n*,**$\boldsymbol{\varepsilon)}$**.** An expression for the ontogenic growth rate of an individual is also constrained by metabolic scaling theory (West et al. 2001):

$g_{ontogenic growth}=w_{0}\varepsilon^{2/3}-w_{1}\varepsilon$ (C-6)

This expression for *g*($\varepsilon)$ is multiplied by *n* to give the contribution of ontogenic growth to *h*(*n*,$\varepsilon):$

$h_{ontogenic growth}=w_{0}n\varepsilon^{2/3}-w_{1}n\varepsilon$ (C-7)

For reasons of scale consistency, the parameter *w*_1_ equals a scale-independent parameter, *w*_10_, divided by ln^2/3^(1/$\beta)$. If, instead, we assumed that *w*_1_ and not *w*_10_ is scale independent, then in the resulting equation for d*E*/dt (Eq. 28 in text), one of the terms, -*w*_1_*E*, scales isometrically with area while the other scales as area/$\ln^{2/3} \left( 1/\beta\right)$ or roughly area/ln^2/3^(area).

Next we turn to the function *q*(*n,*$\varepsilon$), which, when averaged over the structure function, gives d*S*/d*t*.

**Contribution of local extinction to *q*(*n*,**$\boldsymbol{\varepsilon}$**).** We assume that the local extinction of a species in an ecosystem occurs when, within the local community, the last individual in that species dies. The extinction contribution to *q* is then:

$q_{\mathrm{extinction}}=S\frac{dn}{dt}|_{death,n=1}=-\frac{Sd_{0}E/{E_{c}\delta_{n,1}}}{\varepsilon^{1/3}}$ (C-8)

where $\delta_{n,1}$= 1 if *n* = 1 and 0 otherwise.

**Contribution of immigration to *q*(*n*,**$\boldsymbol{\varepsilon}$**).**  Most immigrants will be from species in the meta-community already present in the local community. We assume new species arriving in the local community originate from the relatively rare species in the meta-community and that the metacommunity is static.

The metacommunity species richness, *S*_meta_,_,_ and total abundance, *N*_meta_, together determine $\beta_{\mathrm{meta}}$ (Eq. 6 in main text). From this, the fraction of immigrants that are new species entering the $\varepsilon$ = 1 cohort can be estimated. Using a log-series meta-community SAD, and summing over the high rank (i.e. low abundance) species from the highest ranked to a rank equal to *S*_meta_ – *S*, which by our assumption are the species not already present in the local community, we derive (see SI-D for derivation):

$q_{\mathrm{immigration}}=m_{0}e^{-\mu S-\gamma}$ (C-9)

where ${\mu=ln(1/{\beta_{\mathrm{meta}})/{S_{\mathrm{meta}},}} \beta}_{\mathrm{meta}}$ is calculated from Eq. 6 in main text using the values of *S*_meta_  and *N*_meta_ which in turn are estimated in SI-F, and $\gamma$ is Euler’s constant, ~0.577.

**Contribution of speciation to *q*(*n*,**$\boldsymbol{\varepsilon}$**).**  Absent a more complete understanding of how the speciation rate depends on community variables, we examine two expressions for this rate, both of which are certainly simplifications (see, for example, Smith et al. 2014). In one, the speciation rate is proportional to the total number of species (Rabosky, 2013). Motivation for this comes from the fossil record. Following major extinction events, recovery of a diverse biota generally begins slowly and then accelerates (Kirchner & Weil 1995). The acceleration of diversification at small S is expected if the speciation rate is approximately proportional to species richness. Hence in speciation model 1 we have;

$q_{speciation\_1}$ $=\sigma_{1}\frac{KS}{K+S}$ (C-10)

where *K* is a saturation term.

In an alternative speciation model, each species would have a speciation rate that is proportional to its population size divided by the turnover time of individuals in the species (see, for example, Weiser et al. 2018). In other words, each birth has an equal chance of resulting in a new species, and so species with large *n* and small $\varepsilon$, and thus higher birth rate, have the highest speciation rates:

$q_{speciation\_2}= \frac{{\sigma_{2}b}_{0}nS}{\varepsilon^{1/3}}$ (C-11)

REFERENCES

Kirchner, J. W. & Weil, A. (1998). No fractals in fossil extinction statistics. *Nature* 395, pp. 337–338.

Marba, N., Duarte C. & Agusti, S. (2007). Allometric scaling of plant life history. *PNAS 104*(40), pp.15777-15780.

Niklas, K. (2007). Sizing up life and death. *PNAS 104*(40), pp. 15589-15590.

Rabosky, D.L. (2013) Diversity-dependence, ecological speciation, and the role of competition in macroevolution. *Ann. Rev. Ecol. Evol*. Syst. 44

Weiser, M., Michaletz, S., Buzzard, V., Deng, Y., He, Z., Shen, L., Enquist, B., Wiade, R., Zhou, J. & Kaspari, M. (2018). Toward a theory for diversity gradients: the abundance–adaptation

Hypothesis, *Ecography* 41: 251-264.

West, G., Brown, J. & Enquist, B. (2001). A general model for ontogenic growth. *Nature* *413*, pp. 628-631.

**SI-D. Derivation of the rate of diversification from immigration (first term on right-hand side of Eq. 26 in text).**

The total immigration rate is *m*_0_ immigrant individuals per year entering the local community. Most of these immigrants can be assumed to be individuals from species that are already found in the local community, and in the text we assumed that the probability such an individual is in a particular species with abundance *n* in the local community is equal to the ratio of *n* to the total number of individuals, *N*, in the local community. Implicit, here, is the plausible assumption that species that are abundant in the local community are also abundant in the meta-community, and vice versa.

To estimate the probability an immigrant is in a species that is not already present in the local community, consider the quantity *S*_meta_ – *S*, which is the number of species in the metacommunity that are not present in the local community. Consistent with the previous paragraph, we now assume that these species comprise the rarest species in the metacommunity. If the abundance distribution in the metacommunity is a log-series, as predicted by METE, then the total number of individuals in the *S*_meta_ – *S* rarest species will be given by

$N_{meta, rare}=\frac{S_{\mathrm{meta}}}{\ln\left( 1/{\beta_{\mathrm{meta}}} \right)}\left( \frac{1e^{-{1*\beta}_{\mathrm{meta}}}}{1}+\frac{2e^{-{2*\beta}_{\mathrm{meta}}}}{2}+\frac{3e^{-{3*\beta}_{\mathrm{meta}}}}{3}+\ldots+\frac{\mathrm{Qe}^{-{Q*\beta}_{\mathrm{meta}}}}{Q} \right)$

$\approx\frac{S_{\mathrm{meta}}Q}{ln(1/{\beta_{\mathrm{meta}}})}$ (D-1)

where the second equality is valid if $\beta_{\mathrm{meta}}Q\ll1$and *Q*, the number of individuals in the (*S*_meta_ – *S*)-ranked species in the metacommunity, can be obtained from:

$S_{meta} - S=\frac{S_{\mathrm{meta}}\sum_{n= 1}^{Q} {(e^{-\beta_{\mathrm{meta}}n}}/{n)}}{ln(1/{\beta_{\mathrm{meta}})}}\approx\frac{S_{\mathrm{meta}}(\ln\left( Q \right)+\gamma)}{ln(1/{\beta_{\mathrm{meta}})}}$ . (D-2)

Hence,

$N_{meta,rare}\approx\frac{S_{meta}e^{-\gamma}e^{(1-S/{S_{\mathrm{meta}})ln(1/{\beta_{\mathrm{meta}})}}}}{ln(1/{\beta_{\mathrm{meta}})}}=\frac{S_{meta}e^{-\gamma}e^{(-S/{S_{\mathrm{meta}})ln(1/{\beta_{\mathrm{meta}})}}}}{\beta_{\mathrm{meta}}ln(1/{\beta_{\mathrm{meta}})}}\approx N_{\mathrm{meta}}e^{-\gamma}e^{(-S/{S_{\mathrm{meta}})ln(1/{\beta_{\mathrm{meta}})}}}$ (D-3)

where the last equality follows from Eq. 6 in the text.

Hence, the probability, *p*_new_, that an immigrant is in a new species is given by:

$p_{\mathrm{new}}=\frac{N_{meta,rare}}{N_{\mathrm{meta}}}\approx$ $e^{-\gamma}e^{(-S/{S_{\mathrm{meta}})ln(1/{\beta_{meta})}}}$ (D-4)

and the diversification rate from immigration of new species is given by

$q_{\mathrm{immigration}}= m_{0}e^{-\gamma}e^{-\mu S}$ (D-5)

where

$\mu=\frac{ln(1/{\beta_{\mathrm{meta}})}}{S_{\mathrm{meta}}}$ (D-6)

This results in the first term on the right-hand side of Eq. 26 in the text.

Note that for the parameters in Table 3 of the text, *N*_meta,rare_/*N*_meta_ << 1, implying that at any time step only a very small fraction of new immigrants are likely to be new species near steady state.

**SI-E. Derivations of Eqs. 27-29; 37 in text.**

Here we derive the dependence of the time derivatives of the state variables on the state variables themselves (step 2 of Table 2 in the text). In these calculations, the transition functions are averaged over the static structure function given by Eq. 3 in the main text. We first show how to approximate the relevant integral and sum generally, and then apply our result to the specific transition functions in Eqs. 24-26, as well as to the equation for total biomass in Eq. 37.

Each of the derivations results in averages of the transition functions that differ from what would be obtained if we simply replaced sums of fractional powers of variables with fractional powers of sums of variables. This is a consequence of Jensen’s inequality and the nonlinearity of the dependence of the transition functions on individual metabolic rate $\varepsilon.$

**Approximating the sum and integral**

To take averages of the structure function, we have to approximate terms of the form

$$\begin{aligned} I= \int_{1}^{E_{0}} d\varepsilon\sum_{n=1}^{N_{0}} n^{\nu}\varepsilon^{\sigma}e^{-\lambda_{1}n-\lambda_{2}n\varepsilon} .\#(E-1) \end{aligned}$$

First, consider the integral over $\varepsilon$. We take the upper limit of the integral to infinity, which is a good approximation as long as $\lambda_{2}E_{0}>1$ since the integral is exponentially suppressed and $E_{0}$ is large. This integral is then

$$\begin{aligned} I_{\varepsilon} = \int_{1}^{\infty} d\varepsilon\varepsilon^{\sigma}e^{-\lambda_{2}n\varepsilon},\#\left( E-2 \right) \end{aligned}$$

which is the generalized exponential integral $E_{-\sigma}\left( \lambda_{2}n \right)$. We then assume the sum over $n$ can be approximated by an integral. We pick up a small error term here from the $n=1$ boundary term equal to

$\begin{aligned} \frac{e^{-\lambda_{1}}}{2}E_{-\sigma}\left( \lambda_{2} \right)\approx\frac{e^{-\lambda_{1}}}{2}{\Gamma\left( 1+\sigma\right) \lambda_{2}^{-\sigma-1}},\#\left( E-3 \right) \end{aligned}$

where we have expanded $E_{-\sigma}\left( \lambda_{2} \right)$ for small argument.

Our initial integral can then be written as

$$\begin{aligned} I\approx\int_{1}^{N_{0}} dnn^{\nu}e^{-\lambda_{1}n}E_{-\sigma}\left( \lambda_{2}n \right) .\#\left( E-4 \right) \end{aligned}$$

We now define $t=\lambda_{2}n$ and change variables for the integral to get

$$I\approx\lambda_{2}^{-\nu-1}\int_{\lambda_{2}}^{N_{0}\lambda_{2}} dtt^{\nu}e^{-\frac{\lambda_{1}}{\lambda_{2}} t}E_{-\sigma}\left( t \right)$$

$$\begin{aligned} \approx\lambda_{2}^{-\nu-1}\left( \int_{0}^{\infty} -\int_{0}^{\lambda_{2}} -\int_{{N_{0}\lambda}_{2}}^{\infty} \right)dt t^{\nu}e^{-\frac{\lambda_{1}}{\lambda_{2}} t}E_{-\sigma}\left( t \right) .\#\left( E-5 \right) \end{aligned}$$

The first integral in Eq. E-5 can be solved using Eq. 8.19.25 from the Digital Library of Mathematical Functions (*NIST Digital Library of Mathematical Functions.* http://dlmf.nist.gov/8.19.E25, Release 1.0.28 of 2020-09-15. F. W. J. Olver, A. B. Olde Daalhuis, D. W. Lozier, B. I. Schneider, R. F. Boisvert, C. W. Clark, B. R. Miller, B. V. Saunders, H. S. Cohl, and M. A. McClain, eds.) to relate it to the hypergeometric function. The identity is

$$\begin{aligned} \int_{0}^{\infty} dtt^{b-1}e^{-at}E_{p}\left( t \right)=\frac{\Gamma\left( b \right)\left( 1+a \right)^{-b}}{p+b-1}{}_{2}F_{1}\left( 1,b;p+b;\frac{a}{a+1} \right) ,\#\left( E-6 \right) \end{aligned}$$

where $a>-1$ and $p+b>1$. We set $a=\frac{\lambda_{1}}{\lambda_{2}}$, $b=\nu+1$, and $p=-\sigma$. The first integral in Eq. E-5 then becomes

$$\begin{aligned} \lambda_{2}^{-\nu-1}\int_{0}^{\infty} dtt^{\nu}e^{-\frac{\lambda_{1}}{\lambda_{2}} t}E_{-\sigma}\left( t \right)= \\ \frac{\lambda_{2}^{-\nu-1}\Gamma\left( \nu+1 \right)\left( 1+\frac{\lambda_{1}}{\lambda_{2}} \right)^{-\nu-1}}{\nu-\sigma}{}_{2}F_{1}\left( 1,\nu+1;\nu-\sigma+1;\frac{\lambda_{1}}{\beta} \right) ,\#\left( E-7 \right) \end{aligned}$$

provided that $\frac{\lambda_{1}}{\lambda_{2}}>-1$ and $\nu-\sigma>0$. The first assumption should be satisfied for most ecosystems. The second assumption is required because the integral no longer converges for small $t$ with$\sigma>\nu$. However, we can relax this assumption in our final approximation because this divergence is cancelled out by the integral from 0 to $\lambda_{2}$ in Eq. E-5, and the leading diverging term will come from that integral. Another way to see this is to rewrite Eq. E-4 keeping only the first order term in the $E_{-\sigma}\left( t \right)$ expansion, as that term dominates if $\sigma>\nu$, and then solve $I\approx\lambda_{2}^{-\nu-1}\Gamma\left( 1+\sigma\right)\int_{\lambda_{2}}^{\infty} dt t^{-1-\left( \sigma-\nu\right)}e^{-\frac{\lambda_{1}}{\lambda_{2}} t}.$ We will also take the limit to derive the $\nu=\sigma$ case later.

Since $\frac{\lambda_{1}}{\beta}=1-\frac{\lambda_{2}}{\beta}\approx1$, we can expand ${}_{2}F_{1}$ in Eq. E-7 around $\frac{\lambda_{2}}{\beta}=0$ to get an approximate form for the right hand side. Keeping terms to first order in $\lambda_{2}/\beta$and simplifying gives

$$\begin{aligned} \lambda_{2}^{-\nu-1}\int_{0}^{\infty} dtt^{\nu}e^{-\frac{\lambda_{1}}{\lambda_{2}} t}E_{-\sigma}\left( t \right)\approx\frac{\Gamma\left( \sigma+1 \right)\Gamma\left( \nu-\sigma\right)}{\lambda_{2}^{\sigma+1}\beta^{\nu-\sigma}}\left( 1+\frac{\lambda_{2}\left( \nu-\sigma\right)}{\beta} \right)-\frac{\Gamma\left( \nu+1 \right)}{\beta^{\nu+1}\left( \sigma+1 \right)} .\#\left( E-8 \right) \end{aligned}$$

We now consider the other two integrals in our approximation in Eq. E-4. For the integral from 0 to $\lambda_{2},$ we use the fact that $\lambda_{2}\ll1$ and expand $E_{-\sigma}(t)$ for small argument. This gives the correction term

$$\begin{aligned} \lambda_{2}^{-\nu-1}\int_{0}^{\lambda_{2}} dtt^{\nu}e^{-\frac{\lambda_{1}}{\lambda_{2}} t}E_{-\sigma}\left( t \right)\approx\frac{\Gamma\left( \sigma+1 \right)}{\lambda_{2}^{\sigma+1}\left( \nu-\sigma\right)} .\#\left( E-9 \right) \end{aligned}$$

Finally, for the integral from $\lambda_{2}N_{0}$ to $\infty$, we note that $\lambda_{2}N_{0}\approx S_{0}\gg1$, so we can expand $E_{-\sigma}(t)$ for large argument. This leads to a small correction exponentially suppressed by $e^{-\beta N_{0}}$, which we can safely ignore.

Combining these terms (Eq. E-8, E-9) and including the error from using an integral instead of a sum over $n$ (Eq. E-3) gives a final approximation of

$$\begin{aligned} I\approx\frac{\Gamma\left( \sigma+1 \right)\Gamma\left( \nu-\sigma\right)}{\lambda_{2}^{\sigma+1}\beta^{\nu-\sigma}}\left( 1+\frac{\lambda_{2}\left( \nu-\sigma\right)}{\beta} \right)-\frac{\Gamma\left( \nu+1 \right)}{\beta^{\nu+1}\left( \sigma+1 \right)}-\frac{\Gamma\left( \sigma+1 \right)}{\lambda_{2}^{\sigma+1}}\left( \frac{1}{\nu-\sigma}-\frac{e^{-\lambda_{1}}}{2} \right).\#\left( E-10 \right) \end{aligned}$$

In many cases, only the first term is needed for a good approximation. We will analyze the error terms to determine their relevance below.

For the case where $\nu=\sigma$, we take $\nu-\sigma=\epsilon$ and expand the final approximation Eq. E-10 to first order in $\epsilon$. Taking $\epsilon\to0$ gives the approximation

$$\begin{aligned} I_{\nu=\sigma}=\frac{\Gamma\left( \sigma+1 \right)}{\lambda_{2}^{\sigma+1}}\left( \frac{e^{-\lambda_{1}}}{2}+\ln\left( \frac{1}{\beta} \right)-\gamma\right),\#\left( E-11 \right) \end{aligned}$$

where $\gamma$ is Euler’s constant. In practice, $\left( \frac{e^{-\lambda_{1}}}{2}+\ln\left( \frac{1}{\beta} \right)-\gamma\right)\approx\ln\left( \frac{1}{\beta} \right).$

For the case $\sigma>\nu$, the leading term from Eq. E-10 is

$$\begin{aligned} I_{\sigma>\nu}\approx\frac{\Gamma\left( \sigma+1 \right)}{\lambda_{2}^{\sigma+1}}\left( \frac{1}{\sigma-\nu}+\frac{e^{-\lambda_{1}}}{2} \right),\#\left( E-12 \right) \end{aligned}$$

since the first term will now have $\beta^{\sigma-\nu}$ in the numerator, and $\beta$ is small.

**Error analysis**

There are three sources of error that we have quantified in the above equation.

1. Approximating the hypergeometric function:
   1. $\frac{\Gamma\left( \sigma+1 \right)\Gamma\left( \nu-\sigma+1 \right)}{\lambda_{2}^{\sigma}\beta^{\nu-\sigma+1}}$
   2. $-\frac{\Gamma\left( \nu+1 \right)}{\beta^{\nu+1}\left( \sigma+1 \right)}$
2. Extending the integration bound over $t$ from $\lambda_{2}$ down to 0:
   1. $-\frac{\Gamma\left( \sigma+1 \right)}{\lambda_{2}^{\sigma+1}\left( \nu-\sigma\right)}$
3. Replacing the sum over $n$ with an integral
   1. $\frac{\Gamma\left( \sigma+1 \right)}{\lambda_{2}^{\sigma+1}}\frac{e^{-\lambda_{1}}}{2}$

We can compare the magnitude of these sources of error to the first order term in the approximation to see when they are important to include.

For 1.a., dividing by the first order term in Eq. E-10 gives $\frac{\left( \nu-\sigma\right)\lambda_{2}}{\beta}$, which is small as long as $\lambda_{1}\gg\lambda_{2}$, which is generally true. For 1.b., we get $-\frac{\Gamma\left( \nu+1 \right)}{\Gamma\left( \sigma+2 \right)\Gamma\left( \nu-\sigma\right)} \left( \frac{\lambda_{2}}{\beta} \right)^{\sigma+1}$, which is small provided $\lambda_{1}\gg\lambda_{2}$ and $\sigma>-1$. In most cases then, we can ignore the corrections to the hypergeometric function.

For 2 and 3, doing the same ration with the first order term in Eq. E-10 gives $-\frac{\beta^{\nu-\sigma}}{\Gamma(\nu-\sigma+1)}$ and $\frac{e^{-\lambda_{1}}\beta^{\nu-\sigma}}{2\Gamma\left( \nu-\sigma\right)}$. In both cases, the inverse of the gamma function is order 1 at the largest, so this essentially depends only on $\beta^{\nu-\sigma}$. This can be an important correction if $\nu-\sigma$ is small (or negative) and $\beta$ is large enough; however in many cases we can effectively ignore this term also.

**Derivation of Eq. 27 from Eq. 24**

We start with Eq. 24

$$\begin{aligned} f\left( n,\varepsilon\right)=\left( b_{0}-\frac{d_{0}E}{E_{c}} \right)\frac{n}{\varepsilon^{\frac{1}{3}}}+\frac{m_{0}n}{N}\#\left( E-13 \right) \end{aligned}$$

and Eq. 16

$$\begin{aligned} \frac{dN}{dt}= S\sum_{n,\varepsilon} f\left( n,\varepsilon\right) R\left( n,\varepsilon| S,N,E \right).\#\left( E-14 \right) \end{aligned}$$

The first term in Eq. E-13 corresponds to $\nu=1$ and $\sigma=-1/3$, which plugging into Eq. E-10 gives

$$\begin{aligned} \frac{\left( b_{0}-\frac{d_{0}E}{E_{c}} \right)\Gamma\left( \frac{2}{3} \right)\Gamma\left( \frac{4}{3} \right)}{\lambda_{2}^{2/3}\beta^{4/3}Z} \left( 1+\frac{4\lambda_{2}}{3\beta} \right)-\left( b_{0}-\frac{d_{0}E}{E_{c}} \right)\frac{3}{2\beta^{2}Z}\#(E-15) \end{aligned}$$

We have included the error terms from the hypergeometric function (Error terms 1.a. and 1.b.) because with BCI-like state variables $\frac{\lambda_{2}}{\beta}\approx0.1$, implying that these corrections are needed to get within 10 percent. Since we are subtracting numbers roughly equal in order of magnitude, this 10 percent correction is important.

The second term in Eq. E-13 corresponds to $\nu=1$ and $\sigma=0$, which again plugging into Eq. E-10 gives

$$\begin{aligned} \frac{m_{0}}{{N\lambda}_{2}{\beta Z}}.\#\left( E-16 \right) \end{aligned}$$

Note that this term is actually exactly $m_{0}$ from the constraint $\frac{N}{S}=\sum_{n,\varepsilon} nR$.

Putting Eqs. E-15 and E-16 together gives

$$\frac{dN}{dt}=\left( b_{0}-\frac{d_{0}E}{E_{c}} \right)\frac{S}{Z}\left( \frac{\Gamma\left( \frac{2}{3} \right)\Gamma\left( \frac{4}{3} \right)}{\lambda_{2}^{2/3}\beta^{4/3}}\left( 1+\frac{{4\lambda}_{2}}{3\beta} \right)-\frac{3}{{2\beta}^{2}} \right)+\frac{m_{0}S}{{N\lambda}_{2}\beta Z}.$$

Putting in $\lambda_{2}=S/E,$ $\beta=\frac{S}{N\ln\left( 1/\beta\right)}$, and $Z^{-1}\approx\frac{\lambda_{2}}{\ln\left( 1/\beta\right)}$ along with numerical values for the Gamma functions gives Eq. 27

$$\begin{aligned} \frac{dN}{dt}=\left( b_{0}-\frac{d_{0}E}{E_{c}} \right)\left( \frac{1.21N^{\frac{4}{3}}\ln^{\frac{1}{3}} \left( \frac{1}{\beta} \right)}{E^{\frac{1}{3}}}\left( 1+\frac{4N\ln\left( \frac{1}{\beta} \right)}{3E} \right)-\frac{1.5N^{2}\ln\left( \frac{1}{\beta} \right)}{E} \right)+m_{0}.\#\left( E-17 \right) \end{aligned}$$

**Derivation of Eq. 28 from Eq. 25**

We start with Eq. 25

$$\begin{aligned} h\left( n,\varepsilon\right)=w_{0}{n\varepsilon}^{2/3}-\frac{w_{10}}{\ln^{2/3}(1/{\beta)}}n\varepsilon-\frac{d_{0}E}{E_{c}}n\varepsilon^{2/3}+\frac{m_{0}n}{N}\#\left( E-18 \right) \end{aligned}$$

and Eq. 17

$$\begin{aligned} \frac{dE}{dt}=S\sum_{n,\varepsilon} h\left( n,\varepsilon\right)R\left( n,\varepsilon|S,N,E \right).\#\left( E-19 \right) \end{aligned}$$

The first and third terms in Eq. E-18 have $\nu=1$ and $\sigma=2/3$, which when plugged into Eq. E-10 give

$$\begin{aligned} \left( w_{0}-\frac{d_{0}E}{E_{c}} \right)\frac{1}{Z}\left( \frac{\Gamma\left( 5/3 \right)\Gamma\left( 1/3 \right)}{\lambda_{2}^{5/3}\beta^{1/3}}+\frac{\Gamma\left( 5/3 \right)}{\lambda_{2}^{5/3}}\left( \frac{e^{-\lambda_{1}}}{2}-\frac{1}{1/3} \right) \right)\#\left( E-20 \right) \end{aligned}$$

where we have included the error terms from 2.a. and 3.a. above, which matter as $\nu-\sigma=1/3$, which is small enough to be important.

The second term in Eq. E-18 has $\nu=1$ and $\sigma=1$, which is plugged into Eq. E-11 to give

$$\begin{aligned} -\frac{w_{10}}{\lambda_{2}^{2}Z}\ln^{\frac{1}{3}} \left( 1/\beta\right).\#\left( E-21 \right) \end{aligned}$$

And finally the fourth term in Eq. E-18 has $\nu=1$ and $\sigma=0$, which plugged into Eq. E-10 gives

$$\begin{aligned} \frac{m_{0}}{N\lambda_{2}\beta Z}.\#\left( E-22 \right) \end{aligned}$$

Again, this term is actually exactly $m_{0}$ from the constraint $\frac{N}{S}=\sum_{n,\varepsilon} nR$.

Putting Eqs. E-20, E-21, and E-22 together, again substituting for $\lambda_{2}$, $\beta$, and $Z$ gives Eq. 28

$$\begin{aligned} \frac{dE}{dt}=\left( w_{0}-\frac{d_{0}E}{E_{c}} \right)\left( \frac{2.42 E^{2/3}N^{1/3}}{\ln^{2/3} \left( 1/\beta\right)}-\frac{2.26E^{2/3}S^{1/3}}{\ln\left( 1/\beta\right)} \right)-\frac{w_{10}E}{\ln^{2/3} \left( 1/\beta\right)}+m_{0}.\#\left( E-23 \right) \end{aligned}$$

**Derivation of Eq. 29 from Eq. 26**

We start with Eq. 26

$$\begin{aligned} q\left( n,\varepsilon\right)=m_{0}e^{-\mu S-\gamma}+\sigma_{1}\frac{KS}{K+S}+\sigma_{2}b_{0}S\frac{n}{e^{\frac{1}{3}}}-S\delta_{n,1}\frac{\frac{d_{0}E}{E_{c}}}{\varepsilon^{1/3}}\#\left( E-24 \right) \end{aligned}$$

and Eq. 18

$$\begin{aligned} \frac{dS}{dt}=\sum_{n,\varepsilon} h\left( n,\varepsilon\right)R\left( n,\varepsilon|S,N,E \right).\#\left( E-25 \right) \end{aligned}$$

The first and second terms in Eq. E-24 are constant, so they remain the same under the sum. The third term is similar to the birth term in Eq. E-13 with $\nu=1$ and $\sigma=-1/3,$ thus when plugged into Eq. E-10 gives

$$\begin{aligned} \sigma_{2}b_{0}S\left( \frac{\Gamma\left( 2/3 \right)\Gamma\left( 4/3 \right)}{\lambda_{2}^{2/3}\beta^{4/3}Z}\left( 1+\frac{{4\lambda}_{2}}{3\beta} \right)-\frac{3}{{2\beta}^{2}Z} \right).\#\left( E-26 \right) \end{aligned}$$

For the final term in Eq. E-24, we fix $n=1$ and average $-\frac{Sd_{0}\delta_{n,1}}{\varepsilon^{1/3}}$ over $R\left( 1,\varepsilon|S,N,E \right).$ Here we only need to do the first integral over $\varepsilon$, which we already recognized as $E_{-\sigma}\left( \lambda_{2}n \right).$ Since $n=1$ we can assume a small argument, and expanding gives

$${-d}_{0}S\sum_{\varepsilon} \frac{1}{\varepsilon^{1/3}}R\left( n=1,\varepsilon| S,N,E \right)$$

$$\begin{aligned} \approx-d_{0}S\frac{\Gamma\left( \frac{2}{3} \right)}{\lambda_{2}^{\frac{2}{3}}Z}e^{-\lambda_{1}}.\#\left( E-27 \right) \end{aligned}$$

Finally, combining Eq. E-26 and E-27, substituting for $\lambda_{2}$, $\beta$, and $Z,$ and assuming that $e^{-\lambda_{1}}\approx1$ gives Eq. 29

$$\begin{aligned} \frac{dS}{dt}=m_{0}e^{-\mu S-\gamma}+\sigma_{1}\frac{KS}{K+S}+ \\ \sigma_{2}b_{0}\left( \frac{1.21N^{4/3}\ln^{1/3} \left( 1/\beta\right)}{E^{1/3}}\left( 1+\frac{4N\ln\left( 1/\beta\right)}{3E} \right)-\frac{1.5N^{2}\ln\left( 1/\beta\right)}{E} \right)- \\ \frac{{1.35 d}_{0}}{E_{c}}\frac{S^{\frac{4}{3}}E^{\frac{2}{3}}}{\ln\left( \frac{1}{\beta} \right)}.\#\left( E-28 \right) \end{aligned}$$

**Derivation in Eq. 37**

We start with the first equality in Eq. 37 $\begin{aligned} B=m\left( 1 \right)S\sum_{n,\varepsilon} \varepsilon^{4/3}nR(n,\varepsilon\left| S,N,E \right).\#\left( E-29 \right) \end{aligned}$

Here $\nu=1$ and $\sigma=4/3,$so$\sigma>\nu$ and the leading term comes from $I_{\sigma>\nu}$ in Eq. E-12, which gives

$$\begin{aligned} B=S\frac{\Gamma\left( \frac{7}{3} \right)}{Z\lambda_{2}^{\frac{7}{3}}}\left( 3+\frac{e^{-\lambda_{1}}.}{2} \right).\#\left( E-30 \right) \end{aligned}$$

Again substituting for $\lambda_{2}$ and $Z$, and assuming that $e^{-\lambda_{1}}\approx1$ gives

$\begin{aligned} B=4.17m\left( 1 \right)\frac{E^{\frac{4}{3}}}{S^{\frac{1}{3}}\ln\left( \frac{1}{\beta} \right)} .\#\left( E-31 \right) \end{aligned}$

**SI-F. Justification for the parameter values in Table 3.**

We derive here approximate values of the transition rate parameters that are compatible with the chosen state variables in Table 3: *S* = 320, *N* = 2.3x10^5^, and *E* $\approx$ 2x10^7^ in units such that a tree with 1 cm dbh has a metabolic rate of 1. The values above are intended to approximate the state variables in a generic tropical forest plot much like the 50 ha plot at BCI prior to the flooding of lake Gatun. It is assumed that immigration, not speciation, is the driver of diversification. Different assumptions about state variable values will result in modifications of the rate constants inferred below; however, none of our results in the text will be qualitatively altered if different plausible values are assumed.

We seek approximate values of the parameters *b*_0_, *d*_0_ , *w*_0_, *w*_1_ or *w*_10_, *E*_c_, m_0 ,_ and $\mu$, noting that only the ratio *d*_0_/*E*_c_, and not each separately, actually influences the state variable dynamics in Eqs. 27-29. Three constraints on these parameters arise from the fact that the steady state solutions to Eqs. 27-29 must yield the prescribed static state variables given above. Additional constraints arise from estimations of typical tree growth rates and lifetimes. We will set *b*_0_ equal to *d*_0_.

We set *E*_c_ = 2 x 10^7^ because we know the steady state value of *E* will be close to *E*_c_. The per-capita death rate of trees is given by *d*_0_/$\varepsilon^{1/3}$ and if we assume that average saplings with 1 cm dbh ($\varepsilon$ = 1) are dying at a rate of 0.2/y we obtain *d*_0_ = 0.2. This *d*_0_ value then predicts that trees of average metabolic rate *E*/*N* ~ 100 (dbh = 10 cm) are dying at a rate of ~ 0.04/y; and very large trees with a metabolic rate of 10,000 (dbh = 1 m) are dying at a rate of 0.01/y. These rough estimates are plausible although there is clearly an opportunity here to explore other values.

The growth rate of young trees is dominated by the *w*_0_ parameter, whereas the growth rate of older trees will reflect the values of both w_0_ and $w_{1}= {w_{10}}/{\ln^{2/3} \left( 1/\beta\right).}$ If we assume that a sapling with 1 cm dbh doubles in diameter in 3 or 4 years, while a tree achieves a dbh of 30 cm in 80 years, then we obtain *w*_0_ ~ 1 and *w*_10_ ~ 0.4, where we have used $\ln\left( 1/\beta\right)$= 8.75 These same parameter values imply that it takes ~ 20 years to achieve an average metabolic rate of *E*/*N* ~ 100 (dbh = 10 cm), which is plausible.

There remains estimation of the values of *m*_0_ and $\mu$, which can be determined from the values of *S* and *N* constrained at steady state. Together, these at best suggestive arguments give rise to the values of the rate parameters in Table 3.

We note a cross check on the estimated value of the parameter $\mu$. From it we can estimate the area of the source of immigrants to the BCI plot prior to the flooding of Gatun Lake. The value of $\mu$ in Table 3 implies:

$\frac{ln(1/{\beta_{\mathrm{meta}})}}{S_{\mathrm{meta}}}\approx0.0219$ (F-1)

Assuming *N* scales in proportion to area, and *S* scales according to the static species-area relationship prediction from METE (Harte et al., 2009), Eq. F-1 results in a unique consistent solution in which the effective area of the meta-community is $\approx$128 times the area of the BCI plot (64 km^2^), *N*_meta_ = 128 * 2.30 x 10^5^ = 2.94 x 10^7^, and *S*_meta_ $\approx$ 580, which together give $\ln\left( \frac{1}{\beta_{\mathrm{meta}}} \right)\approx13.4,$ or $\mu=ln(1/{\beta_{\mathrm{meta}})/S_{\mathrm{meta}}}\approx0.0219 as above.$

We note that the value of the migration rate, 500 immigrants recruiting by growth to the 1 cm dbh ($\varepsilon=1)$cohort each year, might appear on the high side. Yet with the parameter values chosen in Table 3, migration only contributes a fraction 0.005 of the entries into the $\varepsilon=1$ cohort; ontogenic growth of non-immigrant seedlings, represented by the *b*_0_ term in Eq. 27, contributes the overwhelmingly dominant source of new recruits.
